# CXCL9 Knockdown Inhibits Tumor Progression and Angiogenesis in Clear Cell Renal Cell Carcinoma via JAK-STAT3 Signaling Pathway

**DOI:** 10.32604/or.2026.084842

**Published:** 2026-10-28

**Authors:** Shi Zong, Kaichen Wang, Yonggang Wang

**Affiliations:** Department of Urology, China–Japan Union Hospital of Jilin University, No. 126, Xiantai Road, Changchun, China

**Keywords:** Clear cell renal cell carcinoma, CXCL9, JAK-STAT3 pathway, epithelial-mesenchymal transition, angiogenesis

## Abstract

**Background:** Advanced clear cell renal cell carcinoma (ccRCC) has a poor prognosis and frequent targeted therapy resistance, which urgently requires novel prognostic biomarkers and therapeutic targets. This study aimed to screen hub genes from our previously constructed ccRCC prognostic gene signature and clarify the oncogenic function and molecular mechanism of CXCL9 in ccRCC progression. **Methods:** Bioinformatic PPI network analysis was performed to screen candidate hub genes; qRT-PCR and IHC verified CXCL9 expression in clinical tissues and cell lines. Lentiviral shRNA was used to stably knock down CXCL9, followed by cell proliferation, apoptosis, migration, invasion and tube formation assays. Rescue experiments, transcriptomic and metabolomic sequencing, as well as subcutaneous xenograft mouse models were applied to validate molecular mechanisms and *in vivo* tumor phenotypes. TCGA-KIRC dataset was analyzed to evaluate correlations between CXCL9 expression, clinical parameters, sunitinib sensitivity and overall survival. **Results:** CXCL9 was determined as a hub gene and substantially upregulated in ccRCC. High CXCL9 expression correlated with distant metastasis, advanced pathological stage, shorter overall survival, and improved sunitinib sensitivity. CXCL9 knockdown suppressed ccRCC malignant phenotypes and angiogenesis, while inducing cell apoptosis. Mechanistically, CXCL9 bound CXCR3 to activate JAK-STAT3, EMT, and inflammatory pathways, which was further validated by multi-omics and rescue assays. *In vivo* assays further confirmed that CXCL9 silencing markedly restrained xenograft tumor growth. **Conclusions:** The CXCL9/CXCR3/JAK-STAT3 cascade serves as a core driver of ccRCC progression. CXCL9 can act as a novel prognostic biomarker and promising therapeutic target for ccRCC.

## 1 Introduction

Clear cell renal cell carcinoma (ccRCC), the main subtype of renal cell carcinoma (RCC) accounting for 70%–75% of cases, contributes to 3% of adult malignancies globally, with advanced cases having a 5-year survival rate below 10% despite improved treatments [1,2,3]. Current treatments for ccRCC primarily involve systemic therapies, including immunotherapy, angiogenesis inhibitors, and mammalian target of rapamycin (mTOR) inhibitors [4]. However, current treatments for ccRCC still experience major challenges, including the absence of specific molecular markers for immunotherapy and frequent treatment resistance, which emphasizes the demand for new therapeutic targets and biomarkers.

Chemokines are pivotal signaling proteins within the cytokine superfamily, widely distributed across diverse cellular contexts, exerting crucial modulatory roles in various physiological and pathological processes [5]. These small secretory proteins demonstrate potent chemotactic properties, orchestrating cellular migration, and positioning [6]. Phosphorylation events on the C-terminal serine/threonine residues further regulate downstream signaling cascades. Among the chemokine receptors, CXCR3 stands out prominently, comprising three distinct spliced variants, namely CXCR3-A, CXCR3-B, and CXCR3-alt [7]. This receptor subtype, classified within the CXC subfamily, demonstrates heightened expression levels in several malignancies, including colon cancer, melanoma, B lymphoma, and breast cancer [8]. Notably, its specific interaction with the ligand CXCL9 governs directional migration of cancer cells, thereby intricately modulating processes underlying cancer invasion and metastasis.

C-X-C motif chemokine ligand 9 (CXCL9), as a chemokine belonging to the CXC family, was initially identified for its function in recruiting immune cells, including T cells and antigen-presenting cells, to inflammation sites [9]. In the context of tumorigenesis, CXCL9 plays a crucial role in tumor immunity and modulation of tumor proliferation, apoptosis, and metastasis, which could be derived from tumor cells [10] and immune cells, including T lymphocytes [11] and macrophages [12]. CXCL9 is a prognostic biomarker for colorectal cancer (CRC) that plays dual roles in tumor progression by activating immune responses and promoting malignant phenotypes. CXCL9, closely associated with mitophagy, blocks autophagic flux by regulating the expression of autophagy-related genes. As a downstream gene modified by ALKBH5, it partially reverses the tumor suppressive effect induced by ALKBH5 knockdown [13]. However, the function and underlying mechanisms of CXCL9 in ccRCC remain poorly understood.

JAK-STAT3 signaling dysregulation has been associated with tumorigenesis and metastasis in various cancers, including ccRCC [14]. Epithelial-mesenchymal transition (EMT) enables epithelial cells to become mesenchymal-like cells, thereby boosting cell migration, invasion, and cancer metastasis via changes in adhesion and cytoskeletal proteins [15]. EMT signaling pathway dysregulation contributes to the invasive phenotype of ccRCC cells, thereby promoting tumor metastasis and therapeutic resistance [16,17]. Dysregulated angiogenesis is a prominent feature in ccRCC, driven by the overexpression of pro-angiogenic factors, including vascular endothelial growth factor (VEGF) [18]. Therefore, targeting angiogenesis has emerged as a promising therapeutic strategy for ccRCC.

Based on our prior bioinformatic research constructing a 44-gene prognostic signature for ccRCC, all 44 signature genes were imported into the STRING database to build a PPI network. Their node degrees within the PPI network were calculated to identify core hub genes, and CXCL9 was ultimately selected for further investigation. This study intends to validate CXCL9’s expression profile and prognostic significance across the TCGA-KIRC cohort and matched clinical ccRCC tissues alongside its associations with clinicopathological traits and tyrosine kinase inhibitor (TKI) responsiveness, characterize CXCL9’s *in vitro* regulatory functions in ccRCC proliferation, apoptosis, migration, invasion and angiogenesis via lentiviral CXCL9 knockdown, delineate the CXCR3-dependent JAK-STAT3 and epithelial-mesenchymal transition (EMT) signaling cascades mediating CXCL9-driven ccRCC malignant phenotypes through rescue assays and multi-omics transcriptomic and metabolomic sequencing, and further confirm CXCL9’s oncogenic activity in subcutaneous xenograft mouse models, with the ultimate goal of uncovering CXCL9’s tumor-promoting function and downstream regulatory axis to identify new biomarkers and therapeutic targets for ccRCC clinical management.

## 2 Materials and Methods

### 2.1 Construction of Protein-Protein Interaction (PPI)

The Search Tool for the Retrieval of Interacting Genes/Proteins (STRING) database (https://string-db.org/), which provides a comprehensive assessment of protein interactions, including both direct (physical) and indirect (functional) associations within a given organism, was used for PPI analysis [19]. The 44 genes previously identified were input into the STRING database to develop the PPI network. Subsequently, Cytoscape software (version 3.3.0; Cytoscape Consortium, San Diego, CA, USA; https://cytoscape.org/) was used to reconstruct the PPI network and analysis [20]. The minimum required interaction score was set to 0.400 (medium confidence), and interactions with a combined score of ≥ 0.4 were retained for construction of the initial PPI network. The initial prognostic-gene network was analyzed in Cytoscape using cytoHubba (version 0.1) and the Degree metric. Separately, MCODE (version 2.0.3) was applied to the downregulated-DEG PPI network with Include Loops = false, Degree Cutoff = 2, Node Score Cutoff = 0.2, Haircut = true, Fluff = false, K-Core = 2, and Max Depth from Seed = 100. The highest-scoring module had an MCODE score of 9.8 and comprised 21 nodes and 98 edges. The connectivity degree of each protein node in the PPI network was computed.

Candidate prioritization: was performed sequentially as follows: (1) The previously established 44-gene signature was derived from an archived cohort of 525 TCGA-KIRC tumor samples with matched overall-survival time and vital-status information. No minimum survival-time threshold was documented for this archived signature-training cohort. Prognosis-associated mRNAs were screened using Cox regression and log-rank *p* < 0.05; genes in the resulting co expression network (|r| >= 0.6 and correlation *p* < 0.05) were ordered by ascending log-rank *p* and optimized by stepwise Bayes discriminant training, yielding the 44-gene signature. No numerical Cox HR cutoff was used; (2) all 44 genes were submitted to STRING, interactions with score > 0.4 were retained, and the resulting network contained 20 connected nodes; the five genes with the highest node degrees were selected; (3) these five genes were assessed by qRT-PCR in 20 paired tumor and adjacent tissues using CONFIRMED PAIRED TEST, with *p* < 0.05 considered significant; (4) In the current CXCL9-specific survival analysis, patients with missing survival information or an overall survival time of 60 days or less were excluded, leaving 507 unique patients. Overall survival (OS) was evaluated after dichotomization at a surv_cutpoint-derived CXCL9 cutoff of 171.7110 (high, *n* = 74; low, *n* = 433); and (5) predicted sunitinib and pazopanib IC50 values were compared between median-defined CXCL9 groups using Wilcoxon rank-sum tests.

Benjamini-Hochberg correction was applied to high-dimensional multiple testing in the transcriptomic differential-expression analysis, functional enrichment analyses, GSEA, and metabolomic feature-wise comparisons. Prespecified comparisons in the cellular and animal experiments and the log-rank survival test were evaluated using unadjusted *p* values. Raw and BH-adjusted *p* values are reported for omics analyses. HR with 95% confidence intervals (CIs) were reported for Cox regression, and log_2_ fold change (FC) and correlation coefficients served as effect sizes. Correlations between CXCL9 and CXCR3, JAK2, JAK3, STAT3, VEGFA, and VEGFR2 were evaluated using Spearman correlation based on log_2_(TPM + 1) values. Transcriptomic log_2_FC values represent sh-CXCL9 relative to sh-NC as estimated by the limma model. Metabolomic log_2_FC values were calculated as log_2_(mean abundance in sh-CXCL9/mean abundance in sh-NC). GSE40435 (GPL10558), comprising 101 ccRCC and 101 non-tumor renal specimens, was used as an external tumor-versus-normal expression-validation cohort. All 202 samples in the archived processed matrix were included. After CONFIRMED NORMALIZATION AND PROBE-SUMMARIZATION METHOD, differential expression was assessed using FDR < 0.05 and absolute fold change > 1.5. CXCL9 was upregulated in ccRCC (logFC = 1.89593916, FDR = 7.78 × 10^−27^, raw *p* = 5.96 × 10^−28^). GSE40435 was not used for external validation of overall survival or predicted TKI sensitivity.

Notably, this database study was exempted from full ethical approval by the Ethics Committee, in accordance with the relevant provisions of Article 32 of the Ethical Review Measures for Life Sciences and Medical Research Involving Human Subjects. The exemption was granted because the data used were publicly accessible and did not include any personally identifiable information.

### 2.2 The Cancer Genome Atlas Kidney Renal Clear Cell Carcinoma (TCGA-KIRC) Dataset Acquisition

The TCGA-KIRC dataset was downloaded from the official TCGA data portal (https://portal.gdc.cancer.gov/) on 31 December 2025. The expression cohort comprised 538 tumor samples and 72 adjacent normal samples. Corresponding clinical variables, including TNM stage, pathological stage, follow-up time, and survival status, were also obtained.

The TCGA-KIRC expression matrix contained 538 tumor aliquots and 72 adjacent non-tumor samples. The 538 tumor aliquots corresponded to 533 patients, including five patients with one additional aliquot each. Sample types were determined using the TCGA sample-type barcode. Before survival analysis, cases with missing survival information or an overall survival time of 60 days or less were excluded, resulting in 507 patients with available survival data. No additional quality-control thresholds based on sequencing depth, missingness, or outlier detection were applied. Because some patients contributed duplicate aliquots, the 512 rows obtained after merging the expression and clinical data represented aliquot-level records rather than 512 independent patients.

The TPM expression matrix comprised 19,938 genes, 538 tumor samples, and 72 adjacent non-tumor samples, with gene symbols used as row identifiers. Before downstream analyses, expression values were transformed using log_2_(TPM + 1). The transformed matrix was used for tumor-stage analysis, survival analysis, and pRRophetic-based drug sensitivity prediction. No additional gene identifier conversion or duplicate-gene aggregation was performed in the downstream scripts. Because all samples were derived from the same TCGA-KIRC cohort, no additional batch-effect correction was applied.

### 2.3 Analysis of the Association between CXCL9 Expression and Tyrosine Kinase Inhibitor (TKI) Sensitivity

Drug response was predicted using pRRophetic (version 0.5.2) and pRRopheticPredict with drug = ‘Sunitinib’ or ‘Pazopanib’, selection = 1, tissueType = ‘allSolidTumors’, and powerTransformPhenotype = TRUE. All other arguments were left at their package defaults. Drug sensitivity prediction was performed using a TPM expression matrix comprising 19,938 genes and 538 TCGA-KIRC tumor aliquots. Tumor samples were retained according to the TCGA sample-type barcodes, while adjacent non-tumor samples and clinical variables were excluded. Before prediction, expression values were transformed using log_2_(TPM + 1), without additional low-expression filtering, scaling normalization, or duplicate-gene aggregation. The pRRopheticPredict function was used to estimate the IC50 values of sunitinib and pazopanib with selection = 1, tissueType = “allSolidTumors”, and power Transform Phenotype = TRUE; all other arguments were maintained at their default values.

### 2.4 Survival Analysis

Patients with missing survival information or an OS time of 60 days or less were excluded. To avoid treating duplicate tumor aliquots as independent observations, one tumor aliquot was retained per patient, with the 01A aliquot preferentially selected when available. The final survival cohort comprised 507 unique patients. The optimal cutoff for CXCL9 expression was determined using survminer::surv_cutpoint in survminer version 0.5.2, with minprop set to its default value of 0.1. On the untransformed TPM-labeled expression scale, the data-derived cutoff was 171.7110, resulting in 74 patients in the high-expression group and 433 patients in the low-expression group. OS was defined as the time from diagnosis to death from any cause or the last follow-up. Kaplan–Meier curves and the log-rank test were used to compare OS between the high- and low-CXCL9-expression groups. Patients with high CXCL9 expression showed shorter OS than those with low CXCL9 expression (apparent log-rank *p* = 0.0061). Consistently, univariable Cox proportional hazards regression showed an increased mortality hazard in the high-expression group (HR = 1.66, 95% CI = 1.15–2.39, *p* = 0.0067). Because the expression cutoff and survival association were evaluated in the same cohort, this analysis was considered exploratory. Multivariable Cox regression was not performed; therefore, the observed association was not adjusted for age, sex, TNM stage, pathological stage, or other clinical variables and should not be interpreted as evidence that CXCL9 is an independent prognostic factor.

### 2.5 Differential Expression and Enrichment Analysis of Transcriptomic Data

The limma package (version 3.62.1) was applied to calculate the log_2_FC, *p*-value, and adjusted *p*-value for each gene based on the FPKM values of sh-CXCL9 and sh-NC groups. Each group contained three biological replicates. FPKM values were transformed as log_2_(FPKM + 1) before differential-expression analysis. The transformed expression matrix was fitted using limma:lmFit; the sh-CXCL9-versus-sh-NC contrast was evaluated using contrasts.fit, and moderated statistics were obtained using eBayes. No voom transformation or additional batch-effect covariate was included. Differentially expressed genes were defined as |log_2_FC| > 1 and Benjamini-Hochberg-adjusted *p* < 0.05.

GO BP enrichment was performed using clusterProfiler package (version 4.14.6) and org.Hs.eg.db (version 3.20.0), with pAdjustMethod = ‘BH’, pvalueCutoff = 0.05, and qvalueCutoff = 0.10. minGSSize was 10 for upregulated genes and 5 for downregulated genes.

### 2.6 Gene Set Enrichment Analysis (GSEA)

All genes were ranked according to their log_2_FC values in the comparison of sh-CXCL9 versus sh-NC. GSEA was performed using the fgsea package (version 1.32.0) and the MSigDB Hallmark collection for Homo sapiens (release 2025.1; h.all.v2025.1.Hs.symbols.gmt). Preranked gene set enrichment analysis was performed using the permutation-based implementation of the fgsea R package. The archived script called fgsea() with nperm = 10,000, which invokes the fgseaSimple algorithm, with gene-set sizes restricted to 15–500 genes and a random seed of 1234. GSEA was performed using the Hallmark gene sets. Gene sets meeting both a nominal *p* value < 0.05 and a Benjamini–Hochberg-adjusted FDR < 0.05 were considered significantly enriched. The EMT gene set had an NES of −1.3082, a nominal *p* value of 0.0804, and an adjusted FDR of 0.5219, and therefore did not meet the predefined significance criteria. The enrichment patterns of canonical Hallmark pathways were assessed between the two groups. GSEA was performed using all 50 Hallmark gene sets from MSigDB version 2025.1. The plotEnrichment function was used to generate enrichment plots and visualize normalized enrichment scores of selected pathways.

### 2.7 Differential Metabolite Screening and Enrichment Analysis of Metabolomic Data

Wilcoxon rank-sum test was conducted to assess the differences in metabolite abundance between the sh-CXCL9 and sh-NC groups. *p* values from metabolite-wise comparisons were adjusted using the Benjamini-Hochberg procedure implemented as p.adjust (method = ‘BH’) in R. The fold change of each metabolite was identified as the ratio of mean abundance between the two groups. The normalized metabolomics data were logarithmically transformed and subjected to unit variance (UV) scaling before Orthogonal Partial Least Squares–Discriminant Analysis (OPLS-DA) was performed using SIMCA 18.0.1. Model performance was evaluated by seven-fold cross-validation, which yielded R2Y = 0.981 and Q2 = 0.829. Model robustness and the risk of overfitting were further assessed using a 200-permutation test. The permutation results were 0/200 for Q2 and 23/200 for R2Y, with regression intercepts of −0.80 and 0.93, respectively. Because each group contained only six biological replicates, the OPLS-DA results were interpreted as exploratory evidence.

In the original analysis, *p* < 0.05 referred to the raw *p* value from the Wilcoxon rank-sum test. Candidate differential metabolites were defined by VIP > 1, raw *p* < 0.05, and |log_2_FC| > 0.5. Benjamini–Hochberg-adjusted *p* values were also calculated for all 1416 metabolites to assess the influence of multiple testing; however, FDR was not used as a criterion in the original candidate selection. These criteria identified 285 candidate differential metabolites, including 237 upregulated and 48 downregulated metabolites, of which 214 also met BH-adjusted *p* < 0.05. We have clarified the distinction between raw and FDR-adjusted *p* values in the revised manuscript. The names of upregulated and downregulated differential metabolites were separately uploaded to the Pathway Analysis module of MetaboAnalyst 6.0 (https://www.metaboanalyst.ca/; accessed on 4 January 2026). The species was set to Homo sapiens, and the KEGG pathway database was selected as the reference database. The input metabolites were standardized using MetaboAnalyst’s built-in name and identifier-matching system. Pathway enrichment analysis and pathway topology analysis were conducted using the platform’s default settings. The upregulated and downregulated differential metabolites were separately uploaded for Kyoto Encyclopedia of Genes and Genomes pathway enrichment in the Pathway Analysis module. All differential metabolites were uploaded in the network analysis module to develop and visualize the metabolite–metabolite interaction network.

### 2.8 PPI and Gene–Metabolite Interaction Networks

Downregulated DEGs were mapped to Homo sapiens in STRING database (version 12.0; accessed 4 January 2026). Only interactions with combined score >= 0.700 were retained, yielding 1237 edges for downstream Cytoscape/MCODE analysis. The MCODE (version 2.0.3) plugin in Cytoscape was used to identify densely connected modules, and the highest-scoring cluster was extracted as the core PPI module. MCODE was applied with Include Loops = false, Degree Cutoff = 2, Node Score Cutoff = 0.2, Haircut = true, Fluff = false, K-Core = 2, and Max Depth from Seed = 100. The highest-scoring module had an MCODE score of 9.8 and contained 21 nodes and 98 edges. Furthermore, the official gene symbols of the downregulated differentially expressed genes, together with the names and log_2_FC values of the differential metabolites, were uploaded to the Network Analysis module of MetaboAnalyst 6.0 (https://www.metaboanalyst.ca/; accessed on 4 January 2026). Input identifiers were standardized and mapped using the platform’s built-in name and identifier-matching system. Gene–metabolite interaction relationships were obtained mainly from the integrated STITCH-based interaction knowledgebase. Only successfully mapped genes and metabolites with known direct interactions were retained, and a gene–metabolite bipartite network was constructed accordingly. The log_2_FC values were used as node attributes.

The integrated network comprised 70 gene nodes, 23 metabolite nodes, and 118 externally retrieved gene-metabolite edges. Edges were not inferred from correlations in the present cohort. No additional topology-based statistical testing or biomarker selection was performed; the network was used as an exploratory visualization of known cross-omic relationships.

### 2.9 Quantitative Real-Time Polymerase Chain Reaction (qRT-PCR)

The tumor and paracancerous normal tissues were collected from 20 patients with ccRCC via surgical resection at the China–Japan Union Hospital of Jilin University between 30 December 2023, and 30 December 2024. All patients signed written informed consent. This research was carried out following the principles of the Declaration of Helsinki. The Ethics Committee of China-Japan Union Hospital of Jilin University approved the experimental procedures (No.2024062606). Patients were enrolled if they had matched fresh tissue samples, intact clinical data and no preoperative anti-tumor treatments, while those with non-ccRCC subtypes, concurrent malignancies, prior anti-tumor therapy, poor-quality specimens or incomplete records were excluded. After surgical resection, tumor tissues were immediately frozen in liquid nitrogen and then stored at −80°C for further examination. The 20 μL qRT-PCR mixture consisted of 10 μL SYBR Premix Ex Taq, 0.4 μL each of forward and reverse primers (10 μmol/L), 2 μL cDNA template and RNase-free water. Thermal cycling included 30 s pre-denaturation at 95°C, 40 cycles of 5 s at 95°C and 30 s at 60°C, followed by melting curve analysis (15 s at 95°C, 1 min at 60°C, gradual heating to 95°C at 0.3°C/s) to confirm specific amplification. Specifically, tissues and cells were subjected to RNA extraction using Trizol reagent (9108, Takara, Kusatsu, Shiga, Japan), followed by cDNA synthesis with the PrimeScript RT reagent kit (RR047A, Takara, Kusatsu, Shiga, Japan). RNA purity was determined by Nanodrop spectrophotometer; qualified RNA samples met the criteria of A260/A280 = 1.8–2.1 and A260/A230 > 1.8. A total of 1 μg qualified total RNA was applied for first-strand cDNA synthesis with PrimeScript RT reagent kit (RR047A, Takara, Kusatsu, Shiga, Japan). Subsequent qRT-PCR amplification was performed on a LightCycler 480 (Roche, Basel, Switzerland) using the SYBR Premix Ex Taq kit (RR420A, Takara, Kusatsu, Shiga, Japan). All procedures were performed under RNase-free conditions. mRNA expression was identified employing the 2^−ΔΔCt^ method. GAPDH was used as the reference gene for normalization. Standard curves generated from serially diluted pooled cDNA confirmed that amplification efficiencies of all primer pairs fell within 90%–110%, meeting the requirements for the 2^^−ΔΔ^Ct method. All primer sequences used for qRT-PCR are summarized in Table 1.

**Table 1:** Primers used for qRT-PCR.

Genes	Primers
CXCL10-F	5′-TCCACGTGTTGAGATCATTGC-3′
CXCL10-R	5′-CGATTCTGGATTCAGACATCTCTTC-3′
CD27-F	5′-AACTGCACCATCACTGCCAAT-3′
CD27-R	5′-CCTGCACTGCCAGCCATT-3′
CXCL9-F	5′-TGCAAGGAACCCCAGTAGTGA-3′
CXCL9-R	5′-CCCTTGGTTGGTGCTGATG-3′
NKG7-F	5′-GAGCGGCAAGATGAGTTTTGAG-3′
NKG7-R	5′-TTCCCGAGGCTCCAGATG-3′
LAG3-F	5′-CAGCTCAATGCCACTGTCACAT-3′
LAG3-R	5′-GGGATCCAGGTGACCCAAA-3′
CXCR3-F	5′-GCAGGTGCCCTCTTCAACAT-3′
CXCR3-R	5′-TAGCGGTCAAAGCTGATGCA-3′
JAK2-F	5′-TGGAGCTTTGGAGTGGTTCTGT-3′
JAK2-R	5′-TCCGCTGGTGGACTTTTACTCT-3′
JAK3-F	5′-GCCGCTTGACAAAGACTACTACGT-3′
JAK3-R	5′-AGATGTTGTCCGAGAGGGATTC-3′
STAT3-F	5′-GAGAAGGACATCAGCGGTAAGAC-3′
STAT3-R	5′-TGATTTCAGCAAATGACATGTTGT-3′
E-Cadherin-F	5′-AAAAGAAGGCTGTCCTTGGC-3′
E-Cadherin-R	5′-GAGGTCTACACCTTCCCGGT-3′
Vimentin-F	5′-AGAGAGAGGAAGCCGAAAGC-3′
Vimentin-R	5′-TCCACTTTCCGTTCAAGGTC-3′
GAPDH-F	5′-GAAGGTGAAGGTCGGAGTC-3′
GAPDH-R	5′-GAAGATGGTGATGGGATTTC-3′

### 2.10 Cell Culture

HK-2 (IMMO-HK-2, IMMO, Xiamen, Fujian, China), 786-O (IMMO-786-O, IMMO, Xiamen, China), A498 (IMMO-A498, IMMO, Xiamen, China), Caki-2 cells (IMMO-Caki-2, IMMO, Xiamen, China), and human umbilical vein endothelial cells (HUVECs) (TCH-C406, HyCyte, Suzhou, China) were procured from the American Type Culture Collection (Manassas, Virginia, USA). HK-2, 786-O, A498, Caki-2 cells, and HUVECs were respectively cultured in Dulbecco’s modified Eagle medium (11965092, Gibco, Grand Island, New York, USA), RPMI-1640 medium (SH30027.01, Cytiva, Marlborough, Massachusetts, USA), Minimum Essential Medium (11095080, Gibco, Grand Island, New York, USA), Caki-2-specialized medium (CM-0326, Procell Inc., Wuhan, Hubei, China) and HUVECs-specialized medium (IMP-H154, Xiamen Immocell Biotechnology Co., Ltd., Xiamen, Fujian, China). The media were supplemented with 10% fetal calf serum (10091148, Gibco, Grand Island, New York, USA), 1% l-glutamine (M11-004, PAA, Pasching, Austria), and 1% penicillin–streptomycin (P11-010, PAA, Pasching, Austria). The cells were maintained at 37°C in a humidified atmosphere containing 5% CO_2_. All cell batches were confirmed mycoplasma-negative by supplier-performed PCR prior to delivery. Cells were routinely visually inspected during culture and experiments for mycoplasma-associated abnormal growth, debris and morphological changes, with no contamination phenotypes observed. All cell lines have been authenticated by STR profiling.

### 2.11 Lentiviral Vectors and Cell Infection

Lentiviral shRNA vectors targeting CXCL9 and scramble negative control (sh-NC) were constructed. The backbone vector was pLV-U6-shRNA-CMV-EGFP-T2A-Puro was provided by JTS Scientific (Wuhan, Hubei, China). JTS Scientific supplied these shRNA and scramble lentiviral vectors. Lentivirus was packaged in 293T cells with a three-plasmid system consisting of transfer plasmid, Gag/Pol and VSV-G helper plasmids. Cell supernatants collected at 48 and 72 h post-transfection were filtered, concentrated by ultracentrifugation and stored at −80°C. Viral titers were determined by qPCR combined with EGFP-positive cell counting, reaching 1.23 × 10^8^ TU/mL for sh-CXCL9 virus and 2.97 × 10^8^ TU/mL for control virus. Three CXCL9 shRNA sequences were designed:

sh-CXCL9#1: 5′-GGTTCTGATTGGAGTGCAA-3′;
sh-CXCL9#2: 5′-CCACCTACAATCCTTGAAA-3′;
sh-CXCL9#3: 5′-CAGCAGATGTGAAGGAACT-3′.

Tumor cells (A498, Caki-2) and HUVECs were seeded 24 h before lentiviral infection. Lentivirus was diluted with complete medium at a multiplicity of infection (MOI) of 10 for ccRCC tumor cells and MOI of 15 for HUVECs. Tumor cells and HUVECs were infected for 48 h under 5 μg/mL polybrene (H9268, Sigma-Aldrich, St. Louis, Missouri, USA), followed by selection with 2 μg/mL puromycin (Sigma-Aldrich, St. Louis, Missouri, USA) for 7 days. The screened cells were considered successful transfections and were used for further functional experiments. The knockdown efficiency of CXCL9 was verified via qRT-PCR and Western blot prior to functional experiments.

### 2.12 Cell Counting Kit-8 (CCK-8) Assay

Transfected cells were collected and resuspended to adjust the cell suspension density to 3.0 × 10^4^ cells/mL. A total of 3 × 10^3^ cells suspended in 100 μL medium were seeded into each well of 96-well plates, with three replicate wells per group. Blank wells containing only 100 μL culture medium without cells were set up for background absorbance subtraction. The cells were cultured at 37°C with 5% CO_2_ for 0, 24, 48 and 72 h. At each predetermined time point, the original culture medium was retained without replacement, and 10 μL CCK-8 reagent was directly added into the existing 100 μL culture medium in each well, followed by gentle mixing. The plates were incubated at 37°C away from light for 1 h. Subsequently, the absorbance at 450 nm was measured using a microplate reader (Infinite M200 Pro, Tecan, Manned Orf, Switzerland).

### 2.13 Western Blot

Cell lysates were prepared by chilling cell culture plates on ice and adding 40 μL of radioimmunoprecipitation assay lysis buffer (P0013B, Beyotime, Shanghai, China) supplemented with protease inhibitor (P1005, Beyotime, Shanghai, China) to each well. The cells were then gently scraped into prechilled tubes and subjected to a 30-min incubation on ice. Afterward, the lysates were centrifuged at 10,000× *g* for 15 min, and the resulting supernatants were carefully collected. Protein concentrations were quantified using a BCA assay (23227, Thermo Fisher Scientific, USA). Briefly, bovine serum albumin (BSA) provided within the kit was used as the protein standard to generate a standard curve with serial dilutions ranging from 0 to 2000 μg/mL. Equal volumes of BCA working reagent and each protein sample were mixed and incubated at 37°C for 30 min before absorbance detection at 562 nm. Based on the quantified protein concentration, a consistent total protein mass of 30 μg per lane was loaded for subsequent SDS-PAGE separation, with equal quantities of protein mixed with 5× protein loading buffer (P0015L, Beyotime, Shanghai, China) and denatured at 95°C for 10 min. Subsequently, proteins were subjected to SDS-PAGE on a 10% polyacrylamide gel, immunoblotting onto PVDF membranes (IPVH00010, Millipore, USA), blocking with 5% skim milk powder (232100, BD Biosciences) diluted in TBST for 1.5 h at 22–25°C. After blocking, membranes were incubated for 12 h at 4°C with gentle shaking in diluted primary antibody solutions prepared with antibody dilution buffer. All primary antibodies were diluted at 1:1000 in TBST containing 1% BSA. For phosphorylated proteins (p-JAK2, p-JAK3, p-STAT3), the corresponding total JAK2, JAK3, and STAT3 protein levels were simultaneously detected on parallel or stripped membranes to normalize phosphorylation intensity; GAPDH was used as the universal internal reference for total protein loading control. After thorough washing, the membranes were probed with Horseradish Peroxidase-conjugated secondary antibodies: Goat anti-Rabbit IgG (111-035-003, Jackson ImmunoResearch, USA) and Goat anti-Mouse IgG (115-035-003, Jackson ImmunoResearch) diluted at 1:5000 in TBST, incubated for 1 h at 22–25°C under continuous shaking. Chemiluminescent signals were visualized using an ECL substrate (34580, Thermo Fisher Scientific), and images were captured using iBright™ CL750 gel documentation imaging system (A44116, Invitrogen, Thermo Fisher Scientific, Waltham, MA, USA). The primary antibodies included anti-CXCL9 (ab137792, Abcam, Cambridge, UK), anti-CXCR3 (ab288437, Abcam, Cambridge, UK), Anti-p-JAK2 (ab32101, Abcam, Cambridge, UK), anti-N-Cadherin (ab76011, Abcam, Cambridge, UK), anti-Bax (ab32503, Abcam, Cambridge, UK), anti-Caspase-9 (ab202068, Abcam, Cambridge, UK), anti-tumor necrosis factor (TNF) alpha (ab183218, Abcam, Cambridge, UK), anti-interleukin (IL)-6 (ab259341, Abcam, Cambridge, UK), anti-VEGFA (ab214424, Abcam, Cambridge, UK), anti-VEGF Receptor 2 (ab221679, Abcam, Cambridge, UK), Anti p-Jak3 (AP0532, ABclonal, Wuhan, Hubei, China), IL-1β Rabbit mAb (A19635, ABclonal, Wuhan, Hubei, China), Ki67 Rabbit mAb (A16919, ABclonal, Wuhan, Hubei, China), Caspase-3 Mouse mAb (A17900, ABclonal, Wuhan, Hubei, China), anti-E-Cadherin (EM0502, HUABIO, Hangzhou, Zhejiang, China), anti-Bcl-2 (ET1702-53, HUABIO, Hangzhou, Zhejiang, China), anti-p-STAT3 (ET1607-40, HUABIO, Hangzhou, Zhejiang, China), anti-Vimentin (ET1610-39, HUABIO, Hangzhou, Zhejiang, China), and anti-VEGF (79478-1, Univ-bio, Beijing, China), total JAK2 (ab108596, Abcam, Cambridge, UK), total JAK3 (A15640, ABclonal, Wuhan, Hubei, China), total STAT3 (ET1607-38, HUABIO, Hangzhou, Zhejiang, China), GAPDH (AF1186, Beyotime, Shanghai, China) (loading control internal reference).

### 2.14 Colony Formation Assay

Briefly, 500 viable sh-NC or sh-CXCL9 cells were seeded into each well of 6-well plates, with three biological replicates per group. Cells were cultured for 14 days to form colonies. After incubation, the cells were washed with phosphate-buffered saline (PBS) and fixed with 4% paraformaldehyde for 15 min at 22–25°C. Subsequently, the fixed cells were stained with crystal violet solution for 20 min. Two blinded independent researchers manually counted colonies containing ≥50 cells under an Olympus CX33 light microscope (Olympus Corporation, Tokyo, Japan), and ImageJ software was used for auxiliary image quantification to reduce counting bias.

### 2.15 Cell Apoptosis Analysis

Cells were detached using 0.25% trypsin-EDTA for 3 min digestion, then harvested via centrifugation at 300× *g* for 5 min; at least 1 × 10^6^ cells per sample were collected. Cell pellets were resuspended in Annexin V-mCherry Binding Buffer. Staining reagents including 1× Annexin V-mCherry and 1× GreenNuc™ Caspase-3 Substrate (C1086M, Beyotime, Shanghai, China) were added, followed by incubation for 15 min in the dark at 22–25°C. Flow cytometry detection was performed on a BD FACSCanto II flow cytometer (338962, BD Biosciences, USA) with mCherry (PE channel) and GreenNuc (FITC channel) fluorescence channels. Single-stained and untreated blank controls were prepared for fluorescence compensation; standard forward scatter/side scatter gating was applied to distinguish intact single cells, early apoptotic and late apoptotic populations. A minimum of 10,000 cellular events were recorded for each sample, and flow data were analyzed using FlowJo V10 software. All flow cytometry analysis experiments were conducted with 3 independent biological replicates.

### 2.16 Scratch Assay for Cell Migration

A scratch assay was performed to assess cell migration. sh-NC and sh-CXCL9 cells were seeded in 6-well plates at a density of 5 × 10^5^ cells per well and cultured until reaching 100% confluence at 24 h post-seeding. Subsequently, scratches were created perpendicular to the horizontal lines using a 200 μL pipette tip. Images were captured to record the initial scratch width. After washing with PBS to remove cell debris, cells were maintained in serum-free medium to eliminate the interference of cell proliferation on wound closure. Images were taken at 0 h and 24 h using an Olympus CX33 inverted light microscope (Olympus Corporation, Tokyo, Japan), and the scratch closure was quantified with ImageJ software v1.54f (National Institutes of Health, Bethesda, MD, USA).

### 2.17 Transwell Assay for Cell Invasion

A transwell assay was employed for cell invasion assessment. Transwell inserts with 8 μm pore-size polycarbonate membranes were applied; the invasion chambers were precoated with Matrigel Basement Membrane Matrix (356234, BD Biosciences, USA), diluted at 1:8 with serum-free medium, with 50 μL diluted matrix gel coated per chamber and polymerized at 37°C overnight. After gel hydration, 2 × 10^5^ Cells suspended in 200 μL serum-free medium were seeded into the upper chamber, while 600 μL complete medium containing 10% FBS was added to the lower chamber as chemoattractant. Cells were incubated for 24 h at 37°C with 5% CO_2_ to allow invasion. After incubation, non-invaded cells on the upper membrane surface were wiped off, and invaded cells were fixed with 4% paraformaldehyde for 15 min, stained via crystal violet staining for 20 min at room temperature. All stained membranes were photographed under an Olympus CX33 inverted light microscope (Olympus Corporation, Tokyo, Japan), and ImageJ v1.54f software was adopted to quantitatively count invaded cells.

The cell migration assay adopted identical seeding cell number, incubation time and culture conditions as the invasion assay; the only difference was that no matrix gel coating was applied to Transwell inserts for migration detection.

### 2.18 Tube Formation Assay

To explore the paracrine effect of ccRCC-derived CXCL9 on angiogenesis, A498 and Caki-2 cells were seeded at 6 × 10^5^ cells per 10 cm culture dish and cultured to 90% confluence, then incubated in serum-free medium for 48 h to collect conditioned medium (CM). Collected supernatant was centrifuged at 300× *g* for 10 min at 4°C, followed by 0.22 μm sterile filtration to remove cell debris. Pre-chilled 48-well plates were coated with 50 μL undiluted Matrigel Basement Membrane Matrix (356234, BD Biosciences, USA) per well, polymerized at 37°C for 30 min. HUVECs were resuspended in the above conditional medium and seeded at a density of 2 × 10^4^ cells per well onto 48-well culture plates precoated with Matrigel Basement Membrane Matrix. Blank medium without tumor cell culture was set as the negative control. Tube formation was observed and photographed at 2, 4 and 8 h under an Olympus CX33 inverted light microscope (Olympus Corporation, Tokyo, Japan). Total tube length and branch number per field were quantified with ImageJ v1.54f software (National Institutes of Health, USA).

### 2.19 Enzyme-Linked Immunosorbent Assay (ELISA)

Serum-free culture supernatants from sh-NC and sh-CXCL9 A498 and Caki-2 cells were harvested after 48 h incubation. Secreted VEGFA (EK1833, Multisciences, Hangzhou, China) and IL-6 levels were quantified using commercial ELISA kits (EK1063, Multisciences, Hangzhou, China) following manufacturer’s instructions. Absorbance at 450 nm with a reference wavelength of 630 nm was measured by a SpectraMax M5 microplate reader (Molecular Devices, USA), and protein concentrations were calculated from standard curves.

### 2.20 Subcutaneous Tumor Model Construction

Caki-2 cells stably expressing sh-NC or sh-CXCL9 were suspended in serum-free medium at a concentration of 2 × 10^6^ cells/mL. Twelve 5-week-old male BALB/c nude mice (purchased from Beijing Vital River Laboratory Animal Technology Co., Ltd., Beijing, China) with consistent body weight (18–20 g) were randomly divided into two groups (*n* = 6 per group) using a random number table; animals were housed under standardized specific pathogen-free (SPF) conditions with 12 h light/dark cycle, constant temperature (22 ± 2°C) and humidity (50% ± 10%), *ad libitum* access to sterilized food and drinking water. Each mouse received a subcutaneous injection of 100 μL cell suspension on the right dorsal flank. Tumor dimensions were measured to calculate tumor volume (volume = length × width^2^). Tumor measurement and histological evaluation were performed by two investigators in a double-blinded fashion. Predefined humane experimental endpoints included a maximum tumor diameter of 15 mm, >20% continuous body weight loss, severe tumor ulceration or obvious poor physical activity. Mice were sacrificed at the predetermined endpoint or 4 weeks after inoculation via gradual CO_2_ inhalation euthanasia with flow rate control to minimize animal distress, consistent with ARRIVE Essential 10 guidelines. Isolated tumors were weighed and processed for subsequent molecular and histopathological analyses. All animal experiments were pre-approved by the Animal Ethics Committee of Jilin University Basic Medical College (No. 2024320), and all experimental reporting strictly followed the ARRIVE Essential 10 guideline.

### 2.21 Hematoxylin and Eosin (H&E) Staining

Tumor tissues were fixed in 4% paraformaldehyde (C104190, Aladdin, Shanghai, China) for 24 h, embedded in paraffin and cut into 4 μm sections. After standard H&E staining reagents (G1005, Servicebio, Wuhan, China), images were captured under an Olympus upright light microscope (BX53, Olympus, Tokyo, Japan) at 4× and 10× magnification. Tumor cellular morphology and karyoplasmic ratio were assessed by two pathologists in a double-blind manner.

### 2.22 Immunohistochemistry (IHC)

Paraffin sections were dewaxed, treated with 3% H_2_O_2_ (H1048, Aladdin, Shanghai, China) to block endogenous peroxidase, and underwent high-pressure antigen retrieval in citrate buffer (pH = 6.0, ZLI-9064, ZSGB-BIO, Beijing, China). Sections were blocked with 5% normal goat serum (SL038, Solarbio, Beijing, China).

Primary antibodies: anti-CXCL9 (ab290643, Abcam, Cambridge, UK), anti-Ki67 (ab15580, Abcam, Cambridge, UK), anti-CD31 (ab182981, Abcam, Cambridge, UK), diluted at 1:1000 and incubated at 4°C overnight. HRP-conjugated goat anti-rabbit secondary antibody (PV-6000, ZSGB-BIO, Beijing, China) was incubated at room temperature for 1 h. DAB chromogenic kit (PV-6000D, ZSGB-BIO, Beijing, China) was used for color development, followed by hematoxylin counterstaining.

Images were acquired at 10× and 40× magnification via Olympus BX53 microscope. Semi-quantitative IRS scoring was performed (IRS ≥ 4 = high expression). ImageJ v1.54f (National Institutes of Health, Bethesda, USA) quantified positive staining area. Ki67 indicated cell proliferation, CD31 reflected microvessel density, and CXCL9 verified gene knockdown efficiency.

### 2.23 TUNEL Assay

Fresh tumor tissues were embedded in OCT compound (4583, Sakura, Tokyo, Japan) and cut into 5 μm-thick cryosections. Slides were fixed with 4% paraformaldehyde for 15 min and permeabilization with 1% Triton X-100 (T434386, Aladdin, Shanghai, China) for 10 min at room temperature. Apoptosis labeling was performed using TUNEL detection kit (C1088, Beyotime, Shanghai, China) with standard kit-provided TdT enzyme and FITC-conjugated dUTP working concentrations; sections were incubated in labeling mixture at 37°C for 60 min in dark. DNase I-treated sections served as positive controls, and TdT-free reaction mix was used for negative control. After labeling, nuclei were counterstained with Hoechst 33342 (H1399, Thermo Fisher Scientific, Waltham, MA, USA). Fluorescence images were captured with a Zeiss LSM 700 laser scanning confocal microscope (Carl Zeiss, Oberkochen, Germany). For quantification, five random non-overlapping fields were blindly selected per section, and at least 2000 total cells were counted to calculate the percentage of TUNEL-positive apoptotic cells.

### 2.24 Statistical Analysis

We processed the data using GraphPad Prism version 8.0 (GraphPad Software, San Diego, CA, USA), and the results are presented as mean ± standard deviation. For comparisons between two groups, two-tailed unpaired or paired Student’s *t*-test was used as appropriate; paired *t*-test was adopted for matched clinical tumor-adjacent tissue pairs. For comparisons among three or more groups, one-way or two-way ANOVA was applied, followed by Tukey’s *post-hoc* multiple-comparison test for multiple comparisons. *p*-values of <0.05 were considered significantly different.

## 3 Results

### 3.1 CXCL9 Was Upregulated in ccRCC

Previously, we constructed a 44-gene prognostic signature via differential expression analysis and multivariate Cox regression from our prior study; detailed Cox hazard ratios, 95% CIs and adjusted *p* values for the top 20 ranked genes are summarized in newly added Table S1. We imported these top 20 prognostic genes into the STRING database to build a PPI network, then used the cytoHubba plugin in Cytoscape to rank all candidates quantitatively by PPI node degree (network connectivity). The full node degree-based ranking list of the 20 genes is visualized in Fig. S1A, where the top five core hub genes with the highest connectivity scores (CXCL9, NKG7, CXCL10, CD27, LAG3) are highlighted. The integrated interaction network of these five hub genes is presented in Fig. 1A. These five genes possessed the highest topological centrality in the gene network, indicating their core regulatory roles during ccRCC initiation and progression. We then performed expression and function validation in ccRCC. As shown in Fig. 1B–F, mRNA expression of CXCL9, NKG7, CXCL10, CD27, and LAG3 was detected using qRT-PCR. Our results revealed a significant upregulation of CXCL9 and NKG7 in ccRCC tissues compared to adjacent normal tissues (full clinical information listed in Table S2). Conversely, CXCL10, CD27, and LAG3 demonstrated no significant differences between tumor and normal tissues. As we know, NKG7 is exclusively expressed in natural killer cells and T cells [21], whereas CXCL9 is expressed by various immune cells and tumor cells [9]. Thus, we selected CXCL9 to investigate its expression and function in ccRCC cells and tumors for further experiments. Fig. 1G,H illustrates that CXCL9 was highly expressed in ccRCC tumor tissues and cells from IHC data and cell mRNA expression analysis.

**Figure 1:**
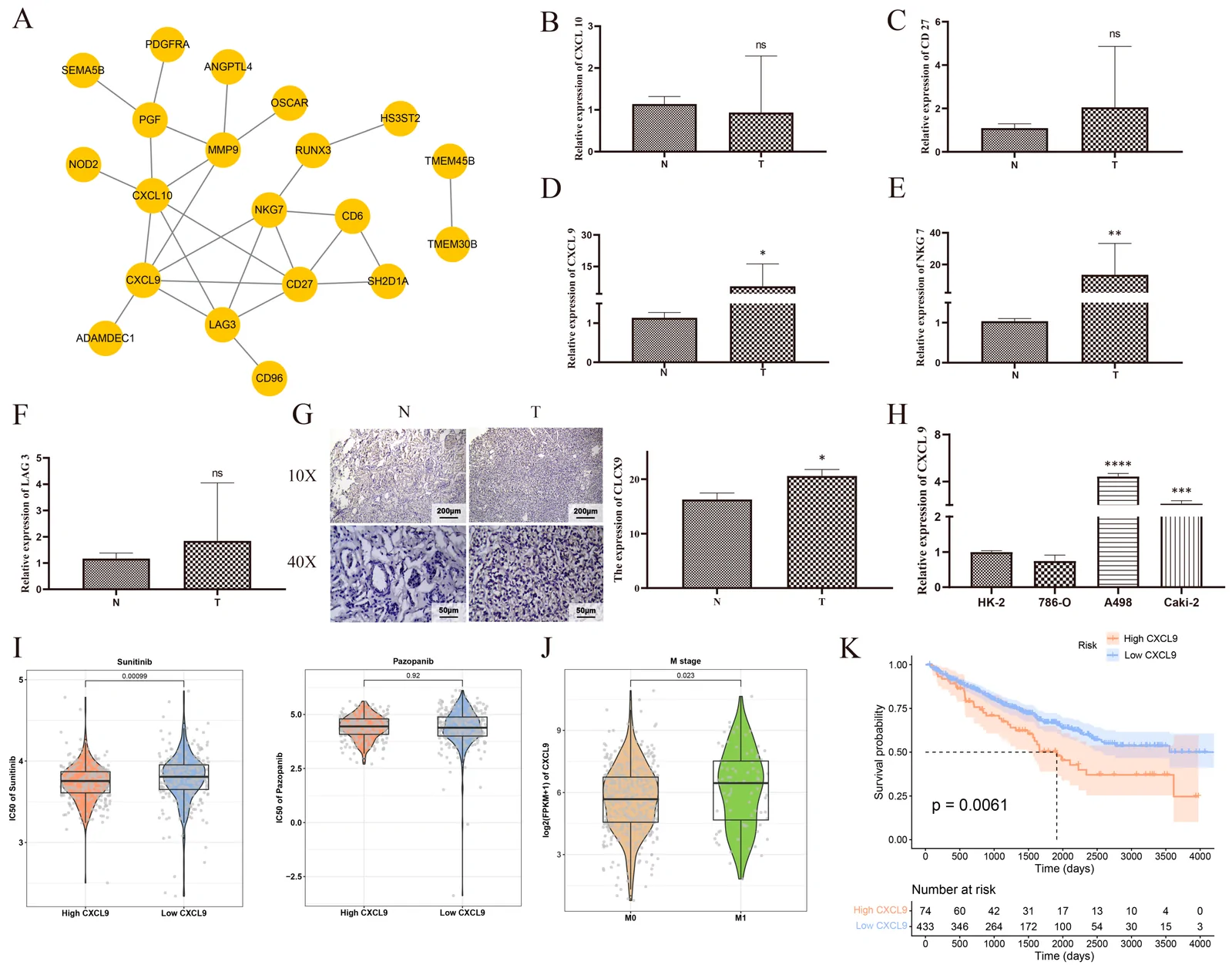
**Expression of C-X-C motif chemokine ligand 9 (CXCL9) in clear cell renal cell carcinoma (ccRCC) tumor tissues and cells.** (**A**) PPI network analysis of differentially expressed genes (DEGs) in ccRCC. (**B**) Relative mRNA expression levels of C-X-C motif chemokine ligand 10 (CXCL10) in paired ccRCC tumor (T) and adjacent normal (N) renal tissues. (**C**) Relative mRNA expression levels of cluster of differentiation 27 (CD27) in paired ccRCC tumor (T) and adjacent normal (N) renal tissues. (**D**) Relative mRNA expression levels of CXCL9 in paired ccRCC tumor (T) and adjacent normal (N) renal tissues. (**E**) Relative mRNA expression levels of natural killer cell granule protein 7 (NKG7). (**F**) Relative mRNA expression levels of lymphocyte-activation gene 3 (LAG3) in ccRCC and adjacent normal tissues detected. (**G**) Expression distribution of CXCL9 in ccRCC tissues and cell lines identified using immunohistochemistry. (Scale bar = 50 μm and 200 μm). (**H**) CXCL9 mRNA expression in normal renal tubular cell line HK-2 and three ccRCC cell lines (786-O, A498, Caki-2). (**I**) Box plots showing the IC50 values of sunitinib and pazopanib in tumor samples between the high and low CXCL9 expression groups. (**J**) CXCL9 expression across different M stages. (**K**) Kaplan–Meier survival curves according to CXCL9 expression in the TCGA-KIRC cohort. Using the data-derived cutoff, the high-CXCL9 group showed shorter OS than the low-CXCL9 group (apparent log-rank *p* = 0.0061; unadjusted HR = 1.66, 95% CI = 1.15–2.39, Cox *p* = 0.0067). All measurements were performed in triplicate. Data are mean ± standard deviation (SD) (*n* = 20). *p < 0.05, **p < 0.01, ***p < 0.001, ****p < 0.0001, ns, no significance.

Subsequently, we explored the correlation between CXCL9 expression and sensitivity to TKI therapy by analyzing the IC50 values of sunitinib and pazopanib. The results revealed that patients with ccRCC with high CXCL9 expression were more sensitive to sunitinib, whereas no significant difference in IC50 values for pazopanib was observed between the high- and low-CXCL9 groups, indicating that CXCL9 expression was not associated with sensitivity to pazopanib (Fig. 1I). Further analysis of CXCL9 expression across different clinical stages of ccRCC revealed that CXCL9 expression was significantly higher in M1-stage tumors than in M0-stage tumors (Fig. 1J). In T stage, N stage, and overall pathological stage, CXCL9 expression demonstrated an upward trend with tumor progression (Fig. S2). Survival analysis revealed that patients with high CXCL9 expression demonstrated significantly shorter overall survival than those with low CXCL9 expression (Fig. 1K).

Furthermore, we performed correlation analyses between CXCL9 and key downstream signaling genes (CXCR3, JAK2, JAK3, STAT3, VEGFA, VEGFR2) based on the TCGA-KIRC transcriptome dataset. The scatter plots confirmed that CXCL9 expression exhibited significant positive correlations with CXCR3, JAK2, JAK3, STAT3, VEGFA and VEGFR2 simultaneously (Fig. S1B–G).

Collectively, this study confirmed that CXCL9 is highly expressed in ccRCC, and its high expression is closely associated with increased sensitivity to sunitinib, advanced tumor progression, and poor prognosis, indicating that CXCL9 may serve as a potential molecular marker for clinical diagnosis, treatment, and prognostic evaluation in ccRCC.

### 3.2 The Oncogenic Function of CXCL9 in ccRCC

To identify the functional importance of CXCL9 on various cellular processes implicated in ccRCC progression, we constructed CXCL9-targeted shRNAs in ccRCC cells. Among three CXCL9-targeted shRNAs, sh-CXCL9 exhibited the highest knockdown efficiency on CXCL9 expression in both A498 and Caki-2 cells (Fig. 2A). Western blot results demonstrated that CXCL9-shRNA2 could effectively downregulate the protein expression of CXCL9 in both cell lines (Fig. 2B). Upon CXCL9 knockdown, we observed a significant decrease in cell proliferation, accompanied by increased apoptosis, impaired colony formation, reduced cell migration and invasion capabilities in ccRCC cells (Fig. 2C–H). Furthermore, CXCL9 knockdown decreased the number and length of HUVEC branches, indicating that CXCL9 could promote angiogenesis *in vitro* (Fig. 2I). ELISA assays revealed that CXCL9 knockdown significantly reduced secreted VEGFA and IL-6 in the culture supernatants of both A498 and Caki-2 cells (Fig. 2J). In conclusion, these results underscored the importance of CXCL9 in regulating key cellular functions associated with ccRCC progression.

**Figure 2:**
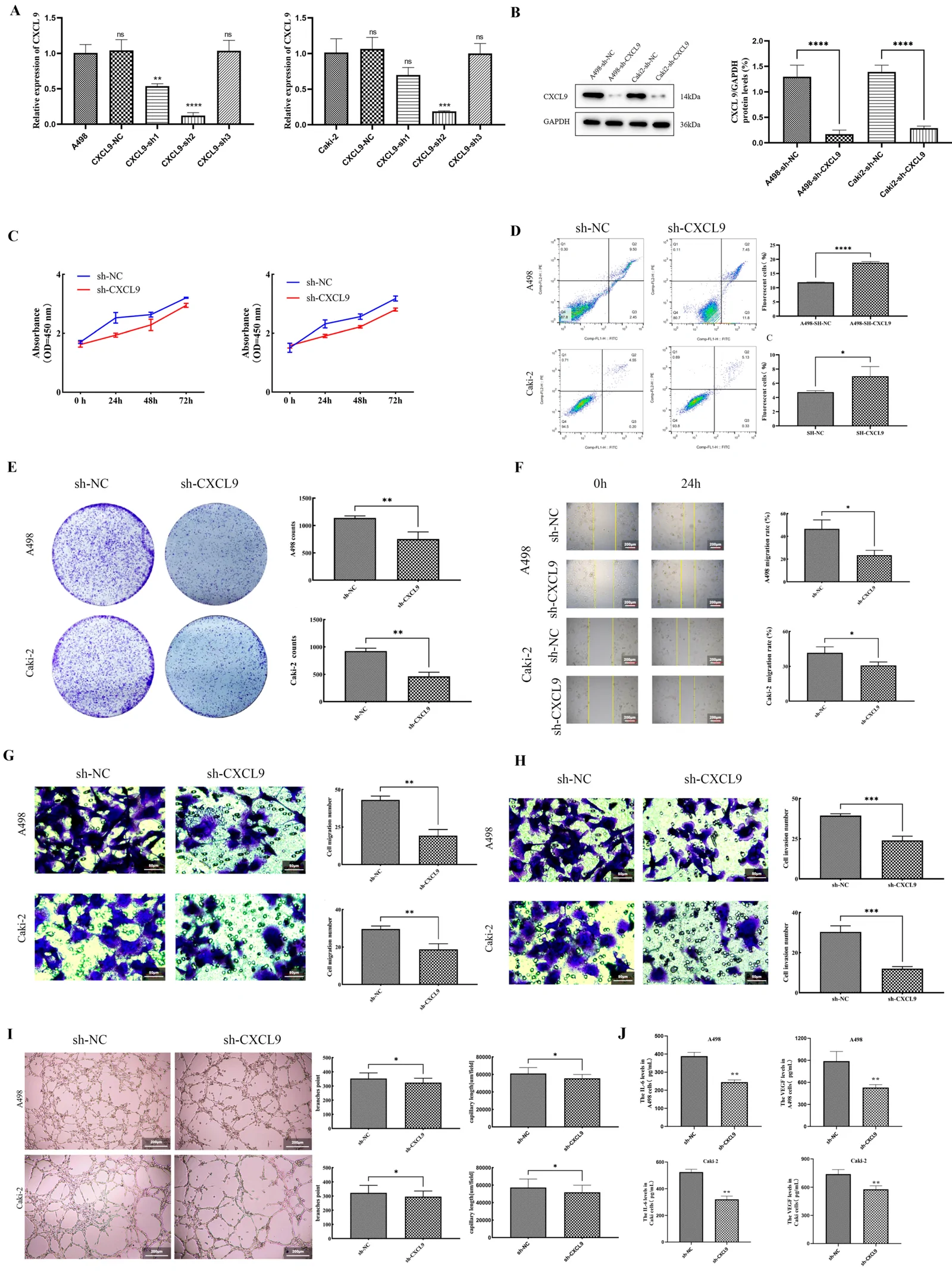
**Functional effects of CXCL9 knockdown on cellular processes in ccRCC.** (**A**) Efficiency of CXCL9 knockdown using shRNA in A498 and Caki-2 cells. (**B**) Validation of CXCL9 knockdown at the protein level in A498 and Caki-2 cells. (**C**–**H**) changes in cell proliferation, apoptosis, colony formation, cell migration (Scale bar = 200 μm), and invasion capabilities (Scale bar = 50 μm) of ccRCC cells after CXCL9 knockdown. (**I**) *In vitro* angiogenesis assay of human umbilical vein endothelial cells (HUVECs) following CXCL9 knockdown, demonstrating branch number and length quantification. (**J**) ELISA detection of secreted VEGFA and interleukin-6 (IL-6) in supernatants from sh-NC and sh-CXCL9 A498 and Caki-2 cells. Data are mean ± SD (*n* = 3). *p < 0.05, **p < 0.01, ***p < 0.001, and ****p < 0.0001, ns, no significance.

### 3.3 CXCL9 Knockdown Inhibits the Activation of JAK-STAT3 and EMT Signaling Pathways

We investigated whether CXCL9 function in ccRCC was associated with JAK-STAT3 and EMT signaling pathways. Our results revealed that CXCL9 knockdown significantly downregulated the mRNA and protein expression of JAK-STAT3 signaling molecules, including JAK2, JAK3, and STAT3, and also reduced the mRNA and protein levels of CXCR3 simultaneously (Fig. 3A,B). Further, CXCL9 knockdown resulted in the inhibition of the EMT-related signaling pathway. The expression of vimentin and N-cadherin was downregulated by sh-CXCL9, whereas E-Cadherin was upregulated (Fig. 3C). Furthermore, CXCL9 knockdown increased the expression of pro-apoptotic proteins, including Bax, Caspase-3, and Caspase-9. Further, anti-apoptotic protein BCL2 was downregulated (Fig. 3D). The expression of inflammation-related cytokines, including TNF-α, IL-1β, and IL-6, was also investigated. The results indicated that they were all downregulated in the Sh-CXCL9 group, indicating the involvement of CXCL9 in the inflammation-related microenvironment in ccRCC. Taken together, CXCL9 exerted its tumorigenic effects in ccRCC mainly via CXCR3-dependent JAK-STAT3 and EMT signaling axes. Furthermore, CXCL9 knockdown promoted tumor cell apoptosis and inhibited the expression of inflammation-related cytokines.

### 3.4 Core Mediating Roles of CXCR3 and JAK-STAT3 in CXCL9-Regulated ccRCC Progression

To further investigate the core roles of CXCR3 and the JAK-STAT3 signaling pathway in regulating ccRCC progression by CXCL9, rescue experiments were performed to verify cellular functions and molecular changes. The results demonstrated that CXCL9 knockdown significantly inhibited the proliferative ability of ccRCC cells (Fig. 4A). Meanwhile, the sh-CXCL9 group showed increased apoptosis and decreased migratory capacity. Conversely, CXCR3 overexpression or JAK-STAT3 activator Coumermycin A1 (CA1) administration significantly reversed the above biological effects, leading to remarkably restored cell proliferation and migration, as well as markedly reduced apoptosis (Fig. 4B,C). As shown in Fig. 4D,E, CXCL9 knockdown significantly downregulated the mRNA and protein expression levels of CXCL9, CXCR3, STAT3, and the EMT marker Vimentin, while significantly upregulating E-Cadherin. CXCR3 overexpression or CA1 treatment effectively reversed the abnormal expression of STAT3, Vimentin, and E-Cadherin. Notably, CXCR3 overexpression markedly increased the mRNA and protein levels of CXCR3 itself, whereas CXCL9 expression remained significantly low in the sh-CXCL9, sh-CXCL9 + oe-CXCR3, and sh-CXCL9 + CA1 groups, with no obvious reversal. Collectively, these results demonstrate that CXCR3 overexpression or JAK-STAT3 pathway activation can effectively rescue the biological dysfunction of ccRCC cells induced by CXCL9 knockdown, thereby further confirming the essential mediating roles of CXCR3 and the JAK-STAT3 signaling pathway in CXCL9-regulated ccRCC progression.

**Figure 3:**
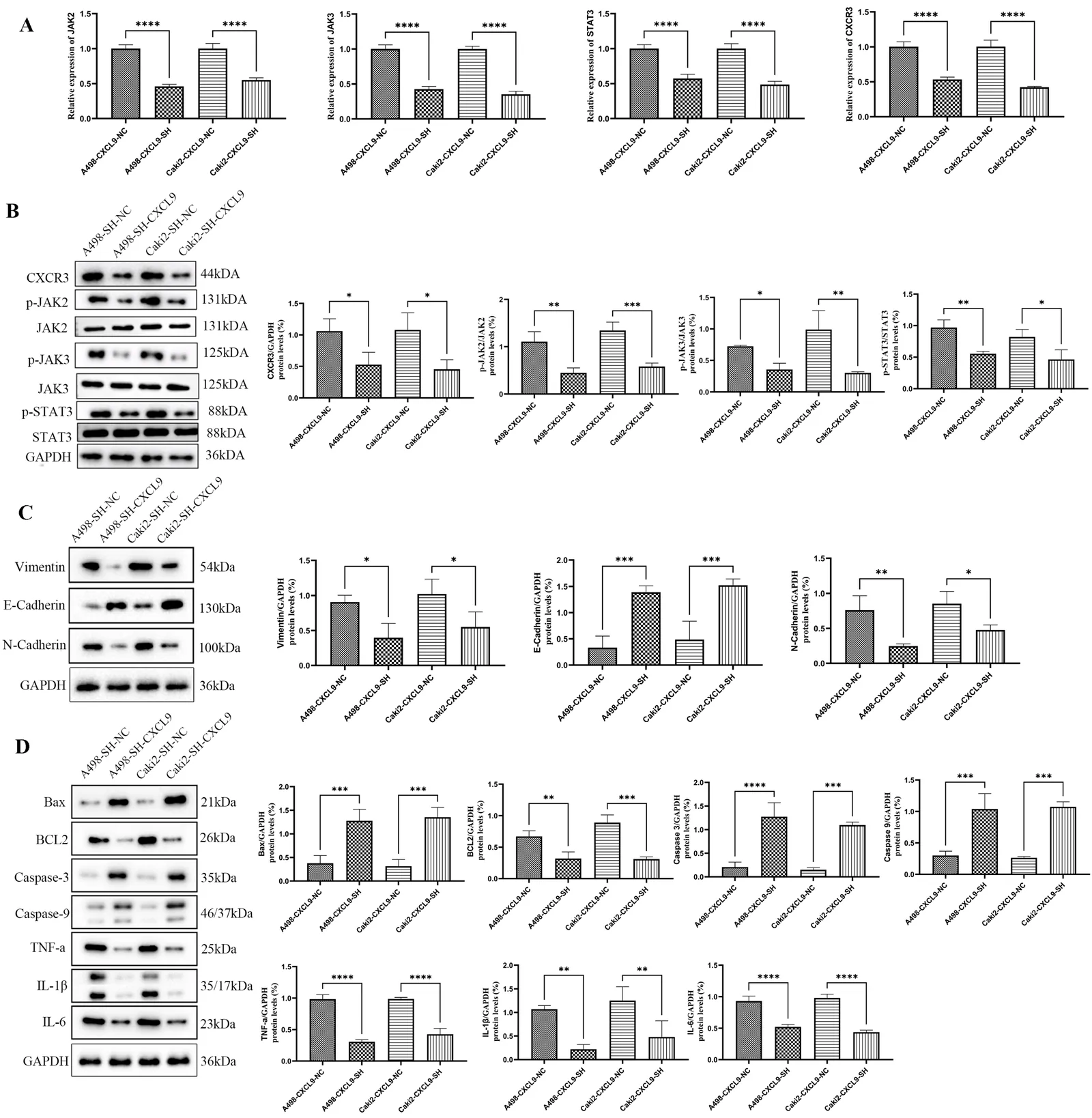
**CXCL9 knockdown inhibited JAK-STAT3 and epithelial-mesenchymal transition (EMT) signaling pathways in ccRCC.** (**A**) Relative mRNA levels of Janus kinase 2 (JAK2), Janus kinase 3 (JAK3), signal transducer and activator of transcription 3 (STAT3), and CXCR3 identified using qRT-PCR. (**B**) Western blot analysis of p-JAK2, p-JAK3, p-STAT3, JAK2, JAK3, STAT3, and CXCR3 protein levels. (**C**) Western blot detection of representative EMT-related proteins. (**D**) Western blot detection of apoptosis- and inflammation-associated proteins, including (Bax), B-cell lymphoma 2 (BCL2), Caspase-3, Caspase-9, tumor necrosis factor-α (TNF-α), IL-1β, and IL-6. Data are mean ± SD (*n* = 3). *p < 0.05, **p < 0.01, ***p < 0.001, and ****p < 0.0001.

**Figure 4:**
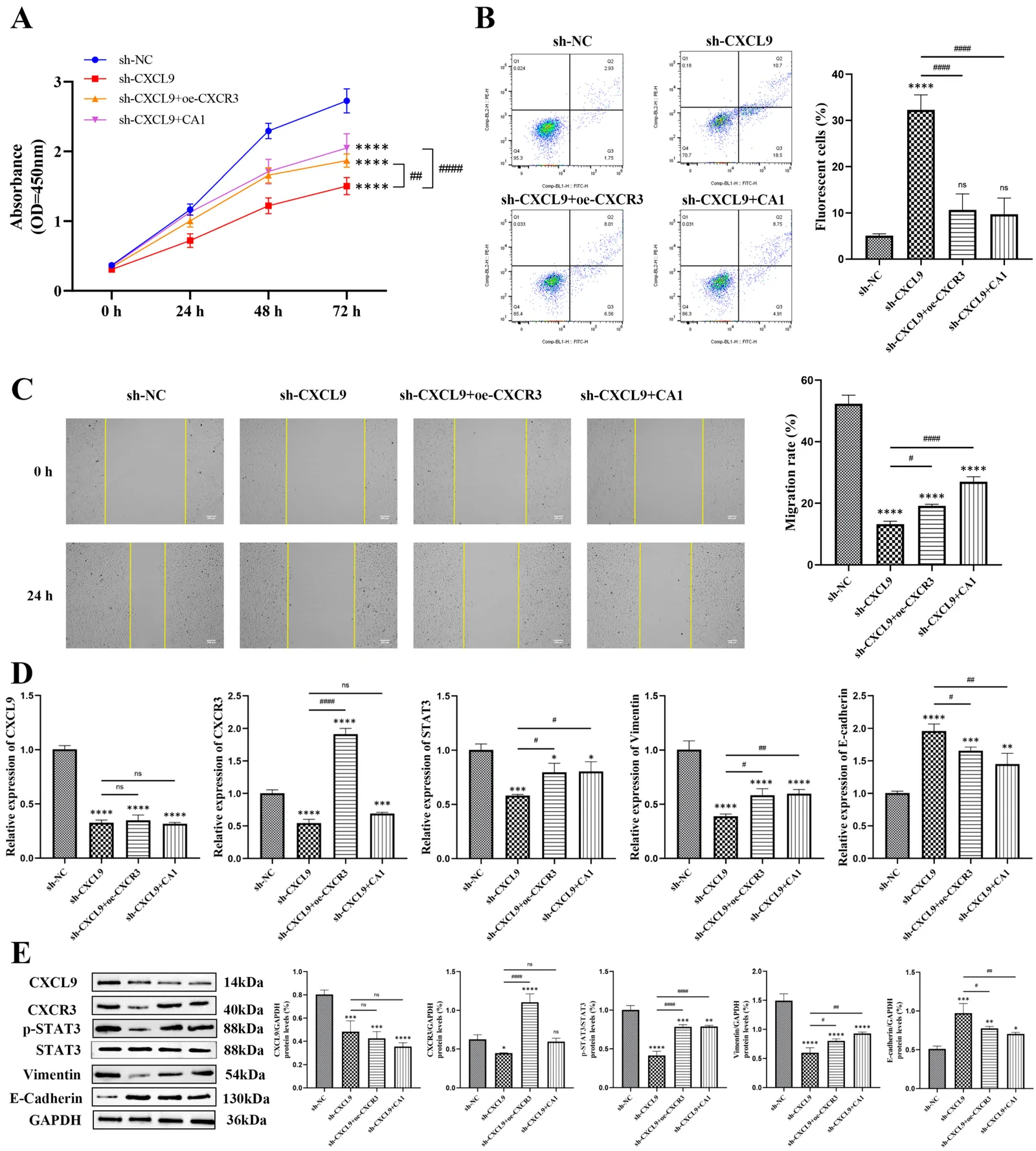
**Functional validation and molecular mechanism underlying the regulatory effect of CXCL9 on ccRCC progression.** (**A**) Cell proliferation detected using CCK8 assay. (**B**) Cell apoptosis measured with flow cytometry. (**C**) Cell migration evaluated using wound-healing assay. (Scale bar = 200 μm). (**D**) Relative mRNA levels of CXCL9, CXCR3, STAT3, E-cadherin, and vimentin identified using qRT-PCR. (**E**) Protein expression of CXCL9, CXCR3, STAT3, E-cadherin, and vimentin examined with Western blotting. Data are mean ± SD (*n* = 3). *p < 0.05, **p < 0.01, ***p < 0.001, ****p < 0.0001 versus the sh-NC group; ^#^*p* < 0.05, ^##^*p* < 0.01, ^####^*p* < 0.0001 versus the sh-CXCL9 group, ns, no significance. Abbreviations: CCK8: cell counting Kit-8.

### 3.5 Transcriptomic and Metabolomic Alterations Triggered by CXCL9 Knockdown in ccRCC Cells

Transcriptome sequencing was performed in this study to further explore the regulatory effects of CXCL9 knockdown on global gene expression in ccRCC cells. Volcano plot analysis identified 1684 upregulated and 1001 downregulated DEGs following CXCL9 knockdown (Fig. 5A). Functional enrichment analysis revealed that upregulated DEGs were mainly enriched in BP related to mitochondrial organization, ribosome biogenesis, and rRNA processing (Fig. 5B), whereas downregulated DEGs were significantly enriched in the mitotic cell cycle, insulin response, and stem cell differentiation (Fig. 5C). Further, GSEA revealed a markedly decreasing trend in the enrichment of EMT-related pathways in the sh-CXCL9 group compared with the sh-NC group (Fig. 5D). Consistent with these findings, the expression levels of JAK1 and STAT3 were both significantly reduced in sh-CXCL9 cells (Fig. 5E), thereby further confirming that CXCL9 silencing effectively inhibits JAK-STAT signaling pathway activation.

Subsequently, metabolomic analysis was conducted to investigate metabolic alterations induced by CXCL9 knockdown. OPLS-DA analysis revealed a clear separation between the sh-CXCL9 and sh-NC groups (Fig. 5F). Metabolites with the top VIP values included 2-[4-(2-Hydroxyethyl)piperazin-4-ium-1-yl] ethanesulfonate, 2′-Hydroxy-4-methylchalcone, and 1-(3,4-dichlorophenyl)-3-[2-(1H-imidazol-4-yl)ethyl] urea (Fig. 5G). In total, 237 metabolites were upregulated and 48 were downregulated (Fig. 5H). Given the limited number of metabolites with an FDR of <0.05, statistical significance was identified using *p*-values. Upregulated metabolites were significantly enriched in glycerophospholipid metabolism and the one-carbon pool by folate (Fig. 5I), whereas downregulated metabolites were involved in amino sugar and nucleotide sugar metabolism, starch and sucrose metabolism, pyrimidine metabolism, and glycolysis/gluconeogenesis (Fig. 5J). Interaction network analysis identified adenine, adenosine, cytidine triphosphate, and glycerol 3-phosphate as hub metabolites (Fig. 5K).

Given that downregulated DEGs were closely associated with the cell cycle, we further constructed a PPI network and identified the highest-scoring cluster using the MCODE method, which included MKI67, AURKA, and KIF20A (Fig. 5L). A combined regulatory network of downregulated DEGs and differential metabolites was then established (Fig. 5M). AURKA interacted with adenosine, and adenosine, oleic acid, and cytidine triphosphate demonstrated extensive connections with downregulated DEGs, thereby highlighting the coordinated transcriptomic and metabolic reprogramming upon CXCL9 silencing.

### 3.6 CXCL9 Deficiency Inhibits Tumor Growth of ccRCC In Vivo

To validate the impact of CXCL9 knockdown on tumor growth and associated functions *in vivo*, we constructed a tumor xenograft model using shCXCL9-transfected ccRCC cells. Our results demonstrated a significant decrease in tumor growth rate and tumor weight in xenograft mouse models upon CXCL9 knockdown (Fig. 6A,B). HE staining results (Fig. 6C) revealed that, in comparison to the sh-NC, the sh-CLCX9 group demonstrated a notable decrease in cell count, nuclear-cytoplasmic ratio, and a marked reduction in the overlapping area of cancer cell nuclei. Ki67 is a well-known proliferation marker for tumors. IHC results indicated significant downregulation of Ki-67, CD31 and CXCL9 in the sh-CXCL9 group (Fig. 6D–F). TUNEL analysis revealed that sh-CXCL9 tumors exhibited a significant increase in apoptotic cell proportion in the tumor, compared with the sh-NC group (Fig. 6G). Taken together, these results demonstrated the critical role of CXCL9 in promoting tumor growth and survival in ccRCC.

**Figure 5:**
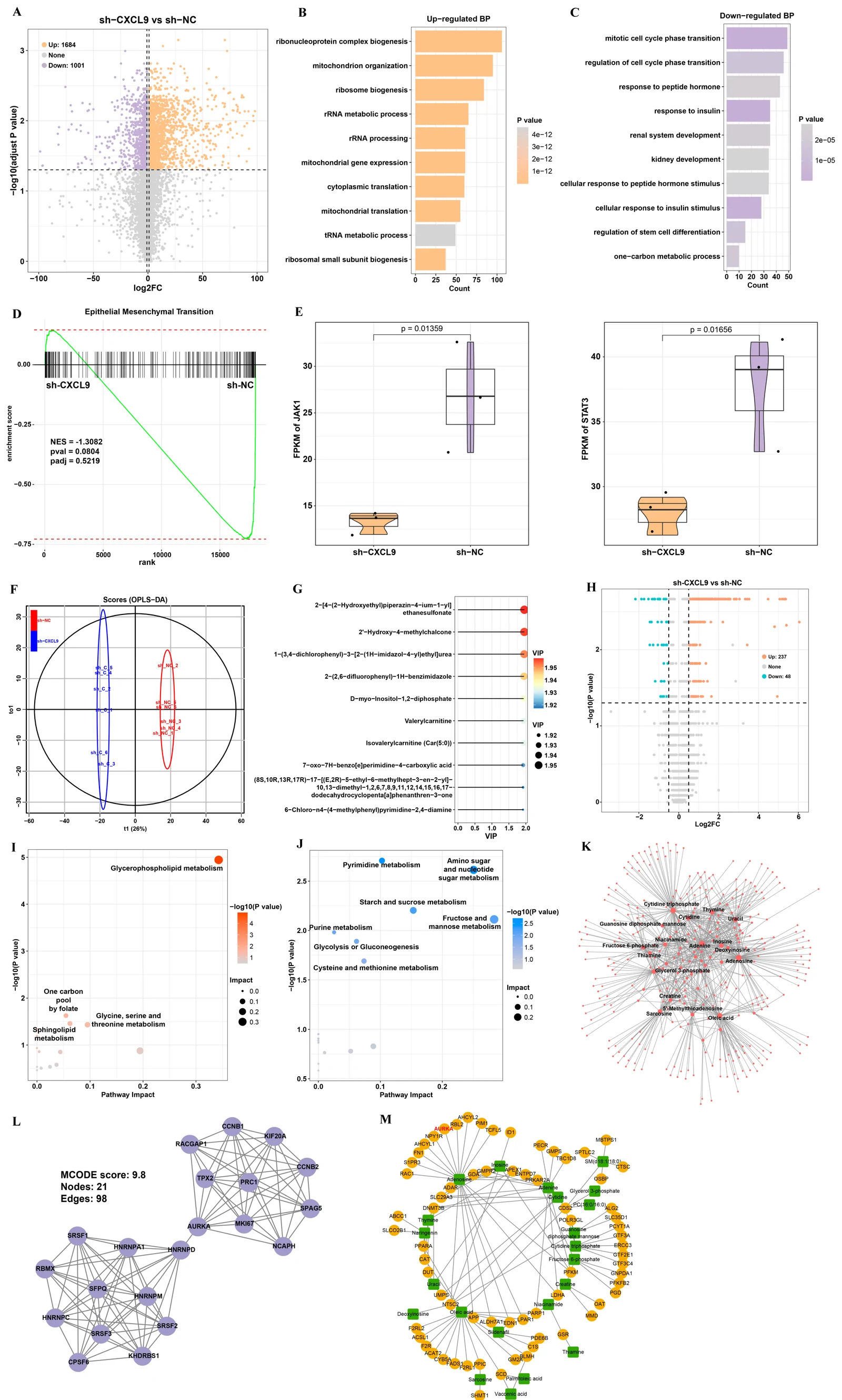
**Transcriptomic, metabolomic, and integrated network analyses upon CXCL9 knockdown.** (**A**) Volcano plot of all DEGs between the sh-CXCL9 and sh-NC groups. (**B**) Top 10 enriched biological processes (BPs) for upregulated DEGs. (**C**) Top 10 enriched BPs for downregulated DEGs. (**D**) Gene set enrichment analysis (GSEA) plot demonstrating the enrichment score of the EMT pathway. (**E**) Box plots illustrating the expression levels of JAK1 and STAT3 between sh-CXCL9 and sh-NC groups. (**F**) OPLS-DA scatter plot of metabolomic profiles. (**G**) Top 10 metabolites ranked by VIP value. (**H**) Volcano plot of altered metabolites between the sh-CXCL9 and sh-NC groups. (**I**) Bubble plot illustrating enriched pathways of upregulated metabolites. (**J**) Bubble plot illustrating enriched pathways of downregulated metabolites. (**K**) Interaction network of differential metabolites. (**L**) Highest-scoring cluster identified with MCODE within the PPI network of downregulated DEGs. (**M**) Integrated interaction network of downregulated DEGs and differential metabolites.

### 3.7 Impact of CXCL9 Knockdown on Intratumoral Signaling Pathways

VEGFA is a classic pro-angiogenic factor expressed by tumor-associated macrophages, which stimulates vascular and lymphatic endothelial cell proliferation and migration and further induces angiogenesis and lymphangiogenesis and contributes to tumor metastasis [22]. CXCL9 knockdown resulted in the downregulated expression of VEGFA and its receptor VEGFR2, indicating that sh-CXCL9 might have an anti-angiogenic capacity for ccRCC (Fig. 7A). The investigation of the JAK-STAT3 pathway also revealed that, following knockdown of CXCL9, JAK2, JAK3, and STAT3 were significantly downregulated compared to the sh-NC group (Fig. 7B,C).

Moreover, the sh-CXCL9 group showed decreased vimentin and N-cadherin, whereas E-Cadherin was significantly increased compared to the sh-NC group (Fig. 8A). CXCL9 knockdown activated the apoptosis-related signaling, including downregulated BCL-2 and upregulated Bax, Caspase-3, and Caspase-9. Finally, we found that CXCL9 knockdown led to the down-regulation of inflammation-related cytokines, TNF-α, IL-1β, and IL-6 (Fig. 8B).

In summary, multiple oncogenic signaling pathways, including angiogenesis, inflammation, and apoptosis signaling, mediated the function of CXCL9.

**Figure 6:**
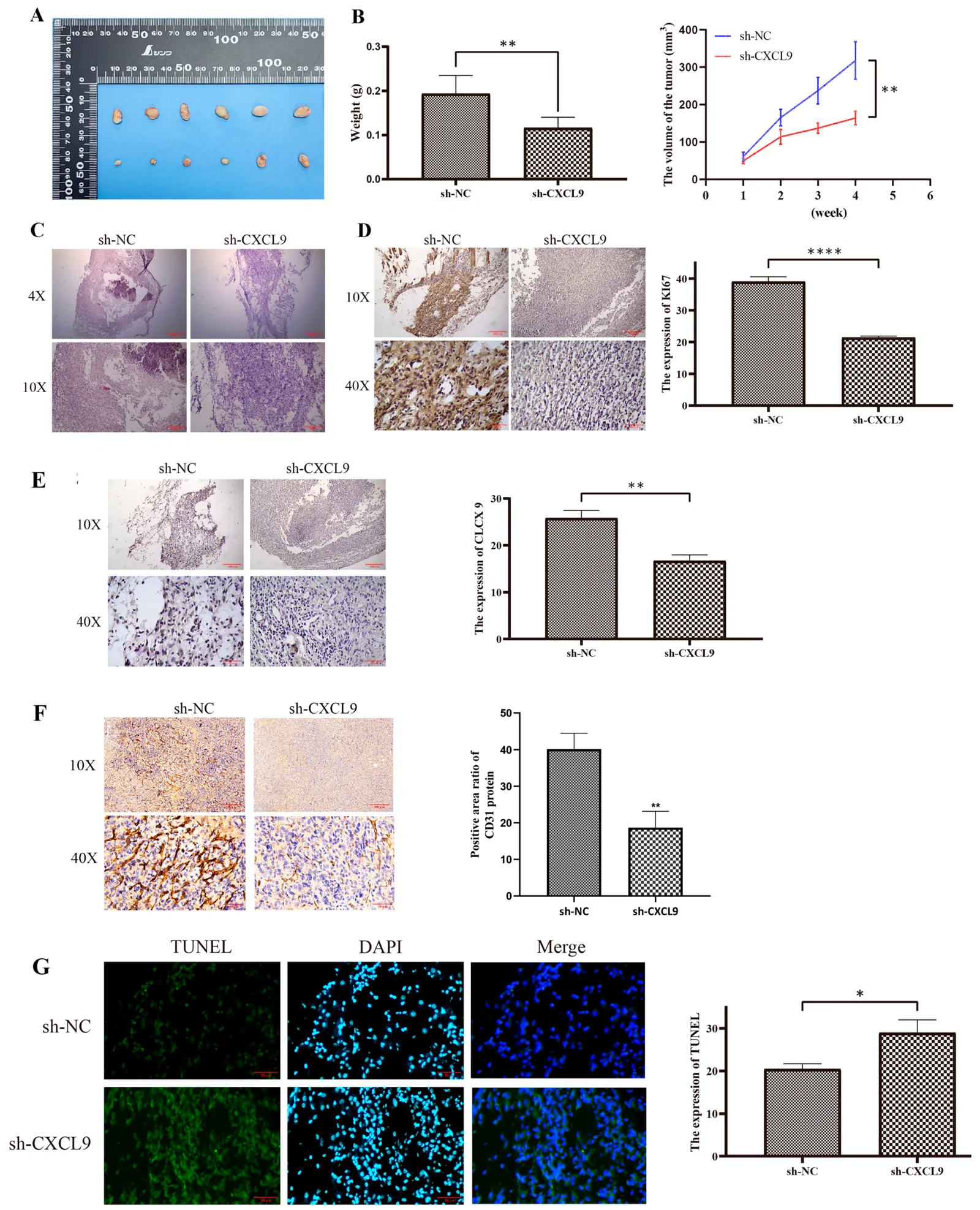
**CXCL9 knockdown inhibited tumor growth and associated functions *in vivo*.** (**A**,**B**) tumor growth rate and tumor weight measurement in ccRCC xenograft mouse models following CXCL9 knockdown. (**C**) Hematoxylin and eosin (HE) staining analysis of morphological alterations in xenograft tumors. (Scale bar = 500 μm and 200 μm). (**D**) Immunohistochemistry of the proliferation marker Ki67. (Scale bar = 50 μm and 200 μm). (**E**) Immunohistochemical staining for CXCL9. (Scale bar = 50 μm and 200 μm). (**F**) Immunohistochemical staining for endothelial marker CD31 to evaluate microvessel density (scale bar = 50 μm and 200 μm). (**G**) TUNEL analysis of apoptotic cell proportion in xenograft tumors after CXCL9 knockdown. (Scale bar = 50 μm). Data are mean ± SD (*n* = 3). *p < 0.05, **p < 0.01, and ****p < 0.0001.

**Figure 7:**
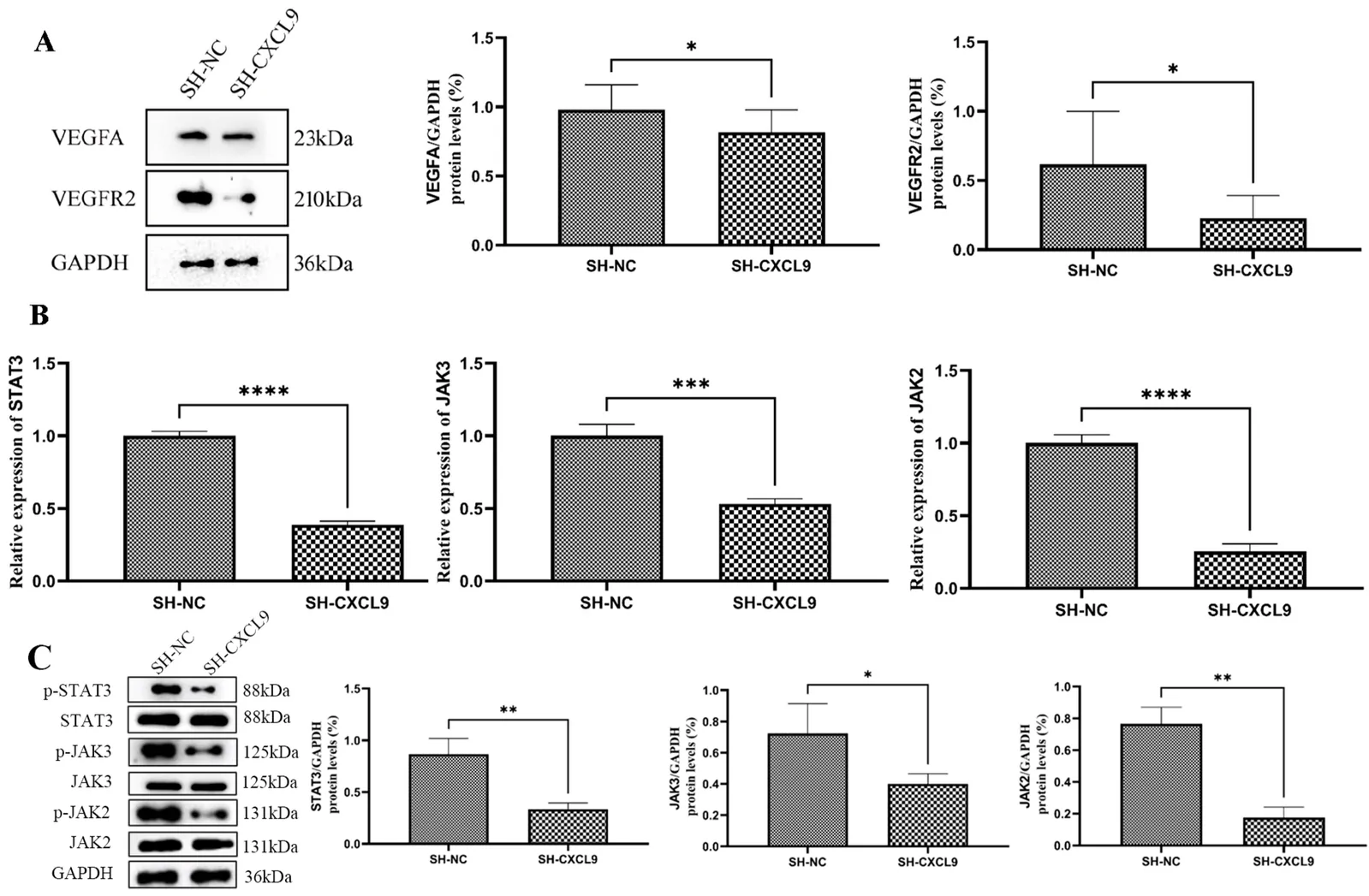
**Impact of CXCL9 knockdown on intratumoral signaling pathways in ccRCC.** (**A**) Western blot analysis of VEGFA and its receptor VEGFR2 expression. (**B**,**C**) detection of STAT3, JAK3, and JAK2 expression using qRT-PCR and Western blot. Data are mean ± SD (*n* = 3). *p < 0.05, **p < 0.01, ***p < 0.001, and ****p < 0.0001.

**Figure 8:**
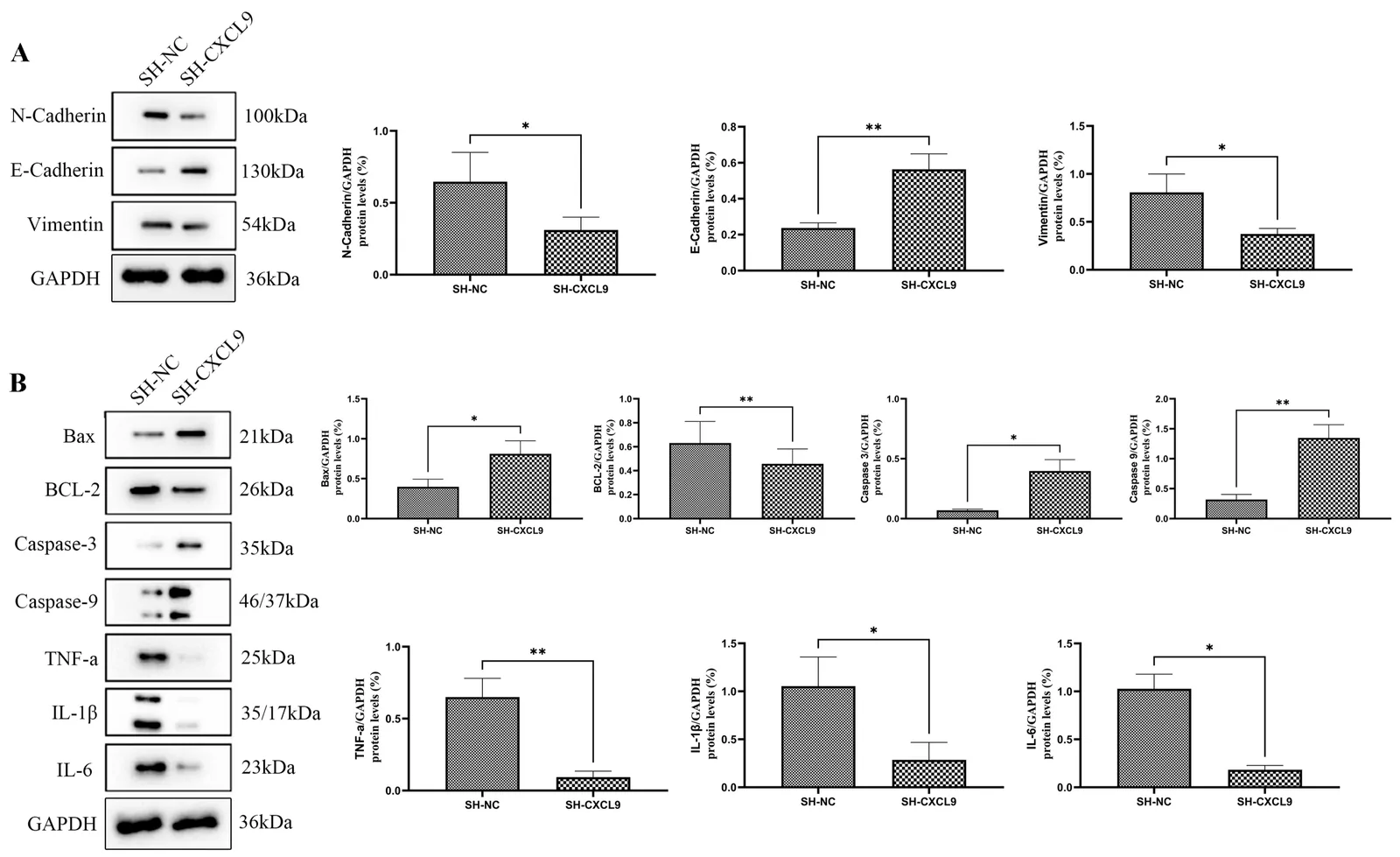
**Influence of CXCL9 knockdown on inflammation and apoptosis signaling in ccRCC.** (**A**) Protein expression of N-cadherin, E-cadherin, and vimentin examined with Western blotting. (**B**) Western blot detection of apoptosis- and inflammation-associated proteins, including Bax, BCL2, Caspase-3, Caspase-9, TNF-α, IL-1β, and IL-6. Data are mean ± SD (*n* = 3). *p < 0.05, and **p < 0.01.

## 4 Discussion

CcRCC is insensitive to radio chemotherapy and prone to targeted drug resistance, necessitating novel prognostic biomarkers and low-toxic natural agents. CDKN3 is aberrantly overexpressed in ccRCC to promote tumor progression by regulating proliferation, metastasis and immune microenvironment [23]. Botanical extracts inhibit PI3K/AKT signaling, suppressing ccRCC cell proliferation, invasion and inducing apoptosis without harming normal renal tubular cells [24]. Despite known oncogenic effects of PI3K/AKT in ccRCC, how CXCL9 drives ccRCC malignancy remains elusive. This study screened hub gene CXCL9 to explore its function in ccRCC progression via the JAK-STAT3 pathway.

Numerous CXC chemokines participate in tumorigenesis and serve as prognostic biomarkers or therapeutic targets across cancers. CXCL5 drives HCC progression via nuclear factor kappa-light-chain-enhancer of activated B cells signaling and predicts poor survival [25]. CXCL13 mediates 5-FU resistance in CRC [26]. High CXCL14 correlates with liver metastasis and worse outcomes in intrahepatic cholangiocarcinoma [27]. Notably, CXCL9 demonstrates dual tumor-regulating effects: it promotes malignancy in HCC, lung cancer, and melanoma by inducing an immunosuppressive microenvironment [28,29,30]; however, it suppresses tumor invasion and improves prognosis in breast and head-and-neck squamous cell carcinoma [31,32].

Against this context, our study identified CXCL9 as a core hub gene together with NKG7, CXCL10, CD27, and LAG3 in ccRCC PPI networks, indicating its vital role in renal tumorigenesis. mRNA and protein detection confirmed CXCL9 upregulation in ccRCC tissues and cells, and high CXCL9 expression correlated with sunitinib sensitivity, tumor progression, and unfavorable patient survival. Subsequent shRNA-mediated CXCL9 knockdown *in vitro* inhibited proliferation, colony formation, migration, invasion, and angiogenesis, while triggering cell apoptosis. These data collectively confirm the oncogenic function of CXCL9 in ccRCC.

Mechanistically, CXCL9 silencing repressed JAK-STAT3 and EMT pathways, accompanied by reduced CXCR3 and pro-inflammatory cytokine expression. Rescue assays further verified that combined CXCR3 overexpression and JAK-STAT3 agonist CA1 treatment reversed the suppressive effects induced by CXCL9 depletion, including rescued malignant phenotypes and the expression of core signaling molecules. Notably, EMT serves as a vital downstream effector of the CXCL9/CXCR3/JAK-STAT3 axis. The altered expression of EMT markers explains the changes in cell migration and invasion [33]. Rescue experiments further confirm that this signaling axis promotes ccRCC metastatic phenotypes by inducing EMT, consolidating the link between molecular regulation and cellular malignant behavior. Consistently, previous reports have validated the CXCL9/CXCR3 axis in regulating JAK/STAT and EMT cascades in triple-negative breast cancer [34]. Our work further establishes this signaling axis as the key driver of ccRCC deterioration.

Transcriptomic analysis revealed suppressed EMT and JAK-STAT signaling after CXCL9 knockdown. Further, integrated transcriptome and metabolome profiling uncovered extensive metabolic reprogramming and remodeled gene–metabolite networks, thereby providing a multiomics landscape to interpret CXCL9-mediated ccRCC progression rarely described in previous renal cancer research.

*In vivo* xenograft models validated the pro-tumor and pro-angiogenic roles of CXCL9. CXCL9 knockdown reduced tumor growth, lowered Ki-67, elevated apoptotic signals, and downregulated VEGFA/VEGFR2. Contrary to its reported anti-angiogenic effect in RCC, colon, and non-small cell lung cancers [35,36,37], CXCL9 facilitates angiogenesis in ccRCC mainly due to dominant expression of pro-tumor CXCR3-A rather than tumor-suppressive CXCR3-B [9]. Distinct CXCR3 subtype expression patterns across tumor types account for CXCL9’s conflicting biological functions.

Several limitations of this study should be noted. Although three CXCL9 shRNAs and partial rescue experiments were implemented in two ccRCC cell lines, further exogenous CXCL9 stimulation, CXCR3/STAT3 pharmacological interventions across additional cell lines are needed to fully confirm the causal CXCL9–CXCR3–JAK–STAT3 cascade linked to EMT and malignant phenotypes. Our *in vivo* experiments relied on subcutaneous xenografts, which cannot recapitulate the orthotopic renal immune microenvironment or spontaneous metastasis of clinical ccRCC. In addition, clinical validation was performed on a small cohort without independent external validation. We only investigated CXCL9 rather than other CXCR3 ligands, and multi-omics analysis lacked proteomic data. These shortcomings are recognized, and relevant experimental improvements will be pursued in future research.

## 5 Conclusion

This study confirmed that CXCL9 is highly expressed in ccRCC, and its upregulation correlates with tumor progression, metastasis, and unfavorable prognosis. Functional assays demonstrated that CXCL9 promotes cell proliferation, migration, invasion, and angiogenesis, and inhibits apoptosis in ccRCC. Mechanistically, CXCL9 modulates the JAK-STAT3, EMT, apoptosis, and inflammatory signaling pathways via binding to CXCR3. Transcriptomics and metabolomics data proved that CXCL9 knockdown triggers gene expression changes and metabolic reprogramming in ccRCC cells. Mouse xenograft models verified that silencing CXCL9 significantly suppresses tumor growth *in vivo*.

In summary, CXCL9 is a crucial oncogenic factor driving ccRCC progression. Targeting CXCL9 and its downstream JAK-STAT3 pathway is expected to become a promising therapeutic approach for ccRCC. This study enriches the molecular mechanism of ccRCC and provides new ideas for clinical targeted therapy.

## Data Availability

The datasets are available from the corresponding author on reasonable request.

## Supplementary Materials

The supplementary material is available online at https://www.techscience.com/doi/10.32604/or.2026.084842/s1.

## Abbreviations

AKT	protein kinase B
BP	biological processes
CA1	coumermycin A1
ccRCC	clear cell renal cell carcinoma
CRC	colorectal cancer
DEGs	differentially expressed genes
DAPI	4′,6-diamidino-2-phenylindole
ECL	enhanced chemiluminescence
EMT	epithelial-mesenchymal transition
FDR	false discovery rate
GSEA	gene set enrichment analysis
HCC	hepatocellular carcinoma
HE	hematoxylin and eosin
HUVECs	human umbilical vein endothelial cells
IC50	half maximal inhibitory concentration
IHC	immunohistochemistry
IL-1β	interleukin-1 beta
IL-6	interleukin-6
KEGG	Kyoto Encyclopedia of Genes and Genomes
KIRC	kidney renal clear cell carcinoma
MCODE	molecular complex detection
NF-κB	nuclear factor kappa-light-chain-enhancer of activated B cells
OPLS-DA	orthogonal partial least squares–discriminant analysis
PI3K	phosphatidylinositol 3-kinase
PPI	protein–protein interaction
PVDF	polyvinylidene difluoride
RCC	renal cell carcinoma
SDS-PAGE	sodium dodecyl sulfate-polyacrylamide gel electrophoresis
SD	standard deviation
STRING	Search Tool for the Retrieval of Interacting Genes/Proteins
TCGA	The Cancer Genome Atlas
TKI	tyrosine kinase inhibitor
TNF-α	tumor necrosis factor alpha
TUNEL	terminal deoxyribonucleotidyl transferase-mediated dUTP nick end labeling
VEGFA	vascular endothelial growth factor A
VEGFR2	vascular endothelial growth factor receptor 2
mTOR	mammalian target of rapamycin

## References

[1] Capitanio U , Bensalah K , Bex A , Boorjian SA , Bray F , Coleman J , et al. Epidemiology of renal cell carcinoma. Eur Urol. 2019; 75( 1): 74– 84. doi:10.1016/j.eururo.2018.08.036. PMC8397918 30243799

[2] Klatte T , Stewart GD . Renal cell carcinoma: Standards and controversies. World J Urol. 2018; 36( 12): 1889– 90. doi:10.1007/s00345-018-2490-5. 30209553

[3] Capitanio U , Montorsi F . Renal cancer. Lancet. 2016; 387( 10021): 894– 906. doi:10.1016/S0140-6736(15)00046-X. 26318520

[4] Rysz J , Ławiński J , Franczyk B , Gluba-Sagr A . Immune checkpoint inhibitors in clear cell renal cell carcinoma (ccRCC). Int J Mol Sci. 2025; 26( 12): 5577. doi:10.3390/ijms26125577. PMC12193005 40565041

[5] Kuo PT , Zeng Z , Salim N , Mattarollo S , Wells JW , Leggatt GR . The role of CXCR3 and its chemokine ligands in skin disease and cancer. Front Med. 2018; 5: 271. doi:10.3389/fmed.2018.00271. PMC6167486 30320116

[6] Tiberio L , Del Prete A , Schioppa T , Sozio F , Bosisio D , Sozzani S . Chemokine and chemotactic signals in dendritic cell migration. Cell Mol Immunol. 2018; 15( 4): 346– 52. doi:10.1038/s41423-018-0005-3. PMC6052805 29563613

[7] Nguyen HT , Hurh S , Nguyen LP , Nguyen TU , Park HK , Seong JY , et al. Functional analysis of CXCR3 splicing variants and their ligands using NanoBiT-based molecular interaction assays. Mol Cells. 2023; 46( 5): 281– 97. doi:10.14348/molcells.2023.2096. PMC10183793 36799104

[8] Ma B , Khazali A , Wells A . CXCR3 in carcinoma progression. Histol Histopathol. 2015; 30( 7): 781– 92. doi:10.14670/HH-11-594. PMC4571436 25663474

[9] Ding Q , Lu P , Xia Y , Ding S , Fan Y , Li X , et al. CXCL9: Evidence and contradictions for its role in tumor progression. Cancer Med. 2016; 5( 11): 3246– 59. doi:10.1002/cam4.934. PMC5119981 27726306

[10] Vandercappellen J , Van Damme J , Struyf S . The role of CXC chemokines and their receptors in cancer. Cancer Lett. 2008; 267( 2): 226– 44. doi:10.1016/j.canlet.2008.04.050. 18579287

[11] Smit MJ , Verdijk P , van der Raaij-Helmer EMH , Navis M , Hensbergen PJ , Leurs R , et al. CXCR3-mediated chemotaxis of human T cells is regulated by a G_i_- and phospholipase C-dependent pathway and not via activation of MEK/p44/p42 MAPK nor Akt/PI-3 kinase. Blood. 2003; 102( 6): 1959– 65. doi:10.1182/blood-2002-12-3945. 12750173

[12] Marcovecchio PM , Thomas G , Salek-Ardakani S . CXCL9-expressing tumor-associated macrophages: New players in the fight against cancer. J Immunother Cancer. 2021; 9( 2): e002045. doi:10.1136/jitc-2020-002045. PMC7919587 33637602

[13] Hu G , Shen S , Zhu M . CXCL9 is a dual-role biomarker in colorectal cancer linked to mitophagy and modulated by ALKBH5. Mol Med Rep. 2025; 32( 1): 188. doi:10.3892/mmr.2025.13553. PMC12076282 40341964

[14] Wang L , Yue Y , Zhang L , Jing M , Ma M , Liu C , et al. PAQR5 inhibits the growth and metastasis of clear cell renal cell carcinoma by suppressing the JAK/STAT3 signaling pathway. Cell Oncol. 2023; 46( 5): 1317– 32. doi:10.1007/s13402-023-00813-w. PMC12974708 37126128

[15] Li D , Xia L , Huang P , Wang Z , Guo Q , Huang C , et al. Heterogeneity and plasticity of epithelial-mesenchymal transition (EMT) in cancer metastasis: Focusing on partial EMT and regulatory mechanisms. Cell Prolif. 2023; 56( 6): e13423. doi:10.1111/cpr.13423. PMC10280150 36808651

[16] Yang C , Zhang Z , Ye F , Mou Z , Chen X , Ou Y , et al. FGF18 inhibits clear cell renal cell carcinoma proliferation and invasion via regulating epithelial-mesenchymal transition. Front Oncol. 2020; 10: 1685. doi:10.3389/fonc.2020.01685. PMC7552945 33117668

[17] Shao I-H , Peng P-H , Wu H-H , Chen J-L , Lai JC-Y , Chang J-S , et al. *RP11-367G18.1* V2 enhances clear cell renal cell carcinoma progression via induction of epithelial-mesenchymal transition. Cancer Med. 2023; 12( 8): 9788– 801. doi:10.1002/cam4.5723. PMC10166984 36847128

[18] Ferician AM , Ferician OC , Nesiu A , Cosma AA , Caplar BD , Melnic E , et al. The mutually mediated chloride intracellular channel protein 1 (CLIC1) relationship between malignant cells and tumor blood vessel endothelium exhibits a significant impact on tumor angiogenesis, progression, and metastasis in clear cell renal cell carcinoma (ccRCC). Cancers. 2022; 14( 23): 5981. doi:10.3390/cancers14235981. PMC9740861 36497464

[19] Szklarczyk D , Nastou K , Koutrouli M , Kirsch R , Mehryary F , Hachilif R , et al. The STRING database in 2025: Protein networks with directionality of regulation. Nucleic Acids Res. 2025; 53( D1): D730– 7. doi:10.1093/nar/gkae1113. PMC11701646 39558183

[20] Kohl M , Wiese S , Warscheid B . Cytoscape: Software for visualization and analysis of biological networks. Methods Mol Biol. 2011; 696: 291– 303. doi:10.1007/978-1-60761-987-1_18. 21063955

[21] Kong G , Song Y , Yan Y , Calderazzo SM , Saddala MS , De Labastida Rivera F , et al. Clonally expanded, targetable, natural killer-like NKG7 T cells seed the aged spinal cord to disrupt myeloid-dependent wound healing. Neuron. 2025; 113( 5): 684– 700.e8. doi:10.1016/j.neuron.2024.12.012. 39809279

[22] Riabov V , Gudima A , Wang N , Mickley A , Orekhov A , Kzhyshkowska J . Role of tumor associated macrophages in tumor angiogenesis and lymphangiogenesis. Front Physiol. 2014; 5: 75. doi:10.3389/fphys.2014.00075. PMC3942647 24634660

[23] Ni C , Guo Z , Bu H , Zhao X , Bao M , Ding L , et al. The role of cyclin-dependent kinase inhibitor 3 in the proliferation and migration of renal cell carcinoma. Chem Biol Interact. 2024; 397: 111092. doi:10.1016/j.cbi.2024.111092. 38825053

[24] Wang A , Yang Y , Chen T , He J , Ling B , Cheng X . Antiproliferative effects of *Trigonostemon* xyphophyllorides on renal cell carcinoma via the PI3K/AKT pathway. Front Pharmacol. 2025; 16: 1594461. doi:10.3389/fphar.2025.1594461. PMC12635619 41282606

[25] Jia X , Wei S , Xiong W . CXCL5/NF-*κ*B pathway as a therapeutic target in hepatocellular carcinoma treatment. J Oncol. 2021; 2021: 9919494. doi:10.1155/2021/9919494. PMC8184336 34194499

[26] Zhang G , Luo X , Zhang W , Chen E , Xu J , Wang F , et al. CXCL-13 regulates resistance to 5-fluorouracil in colorectal cancer. Cancer Res Treat. 2020; 52( 2): 622– 33. doi:10.4143/crt.2019.593. PMC7176956 32019285

[27] Miyata T , Yamashita YI , Yoshizumi T , Shiraishi M , Ohta M , Eguchi S , et al. *CXCL12* expression in intrahepatic cholangiocarcinoma is associated with metastasis and poor prognosis. Cancer Sci. 2019; 110( 10): 3197– 203. doi:10.1111/cas.14151. PMC6778649 31361379

[28] Liu R-X , Wei Y , Zeng Q-H , Chan K-W , Xiao X , Zhao X-Y , et al. Chemokine (C-X-C motif) receptor 3-positive B cells link interleukin-17 inflammation to protumorigenic macrophage polarization in human hepatocellular carcinoma. Hepatology. 2015; 62( 6): 1779– 90. doi:10.1002/hep.28020. 26235097

[29] Nakanishi T , Imaizumi K , Hasegawa Y , Kawabe T , Hashimoto N , Okamoto M , et al. Expression of macrophage-derived chemokine (MDC)/CCL22 in human lung cancer. Cancer Immunol Immunother. 2006; 55( 11): 1320– 9. doi:10.1007/s00262-006-0133-y. PMC11030788 16453150

[30] Amatschek S , Lucas R , Eger A , Pflueger M , Hundsberger H , Knoll C , et al. CXCL9 induces chemotaxis, chemorepulsion and endothelial barrier disruption through CXCR3-mediated activation of melanoma cells. Br J Cancer. 2011; 104( 3): 469– 79. doi:10.1038/sj.bjc.6606056. PMC3049560 21179030

[31] Hsin L-J , Kao H-K , Chen I-H , Tsang N-M , Hsu C-L , Liu S-C , et al. Serum CXCL9 levels are associated with tumor progression and treatment outcome in patients with nasopharyngeal carcinoma. PLoS One. 2013; 8( 11): e80052. doi:10.1371/journal.pone.0080052. PMC3836991 24278236

[32] Ruiz-Garcia E , Scott V , Machavoine C , Bidart JM , Lacroix L , Delaloge S , et al. Gene expression profiling identifies Fibronectin 1 and CXCL9 as candidate biomarkers for breast cancer screening. Br J Cancer. 2010; 102( 3): 462– 8. doi:10.1038/sj.bjc.6605511. PMC2822945 20068563

[33] Wu Y , Terekhanova NV , Caravan W , Naser Al Deen N , Lal P , Chen S , et al. Epigenetic and transcriptomic characterization reveals progression markers and essential pathways in clear cell renal cell carcinoma. Nat Commun. 2023; 14( 1): 1681. doi:10.1038/s41467-023-37211-7. PMC10042888 36973268

[34] Wu L , Sun S , Qu F , Sun M , Liu X , Sun Q , et al. CXCL9 influences the tumor immune microenvironment by stimulating JAK/STAT pathway in triple-negative breast cancer. Cancer Immunol Immunother. 2023; 72( 6): 1479– 92. doi:10.1007/s00262-022-03343-w. PMC10992228 36472587

[35] Zhang R , Tian L , Chen LJ , Xiao F , Hou JM , Zhao X , et al. Combination of MIG (CXCL9) chemokine gene therapy with low-dose cisplatin improves therapeutic efficacy against murine carcinoma. Gene Ther. 2006; 13( 17): 1263– 71. doi:10.1038/sj.gt.3302756. 16672984

[36] Pan J , Burdick MD , Belperio JA , Xue YY , Gerard C , Sharma S , et al. CXCR3/CXCR3 ligand biological axis impairs RENCA tumor growth by a mechanism of immunoangiostasis. J Immunol. 2006; 176( 3): 1456– 64. doi:10.4049/jimmunol.176.3.1456. 16424173

[37] Addison CL , Arenberg DA , Morris SB , Xue YY , Burdick MD , Mulligan MS , et al. The CXC chemokine, monokine induced by interferon-gamma, inhibits non-small cell lung carcinoma tumor growth and metastasis. Hum Gene Ther. 2000; 11( 2): 247– 61. doi:10.1089/10430340050015996. 10680839

