# Supplementary material for: CXCL9 Knockdown Inhibits Tumor Progression and Angiogenesis in Clear Cell Renal Cell Carcinoma via JAK-STAT3 Signaling Pathway
Source: Oncol Res. 2026 Oct 28;34(11):25. doi: 10.32604/or.2026.084842 (PMC13732337; doi:10.32604/or.2026.084842)
Supplement: Supplementary file 1 [file OncolRes-34-84842-s001.zip › TSP_OR_84842-s001.docx]

**Figure S1. Bioinformatic analyses based on the TCGA-KIRC cohort.** (A) Ranking of the top 20 prognostic genes according to protein‑protein interaction (PPI) node degree calculated using cytoHubba. (B) Scatter plot showing the positive correlation between C‑X‑C motif chemokine ligand 9 (CXCL9) and C‑X‑C motif chemokine receptor 3 (CXCR3). (C) Scatter plot showing the positive correlation between CXCL9 and Janus kinase 2 (JAK2). (D) Scatter plot showing the positive correlation between CXCL9 and Janus kinase 3 (JAK3). (E) Scatter plot showing the positive correlation between CXCL9 and signal transducer and activator of transcription 3 (STAT3). (F) Scatter plot showing the positive correlation between CXCL9 and vascular endothelial growth factor A (VEGFA). (G) Scatter plot showing the positive correlation between CXCL9 and vascular endothelial growth factor receptor 2 (VEGFR2). (n = 538).

**Figure S2: Correlation between CXCL9 expression and clinical stages in the TCGA-KIRC cohort.** (A) CXCL9 expression across different T stages. (B) CXCL9 expression across different N stages. (C) CXCL9 expression across different pathological stages. Data are mean ± SD (n = 538).

**Figure 2B-CXCL9**

62kDa

115kDa

240kDa

140kDa

15kDa

27kDa

31kDa

50kDa

80kDa

14kDa

14kDa

14kDa

**Figure 2B-GAPDH**

240kDa

140kDa

115kDa

80kDa

36kDa

270kDa

175kDa

130kDa

95kDa

66kDa

52kDa

36kDa

62kDa

6.5kDa

37kDa

30kDa

36kDa

15kDa

27kDa

31kDa

50kDa

16kDa

**Figure 3B-CXCR3**

15kDa

27kDa

31kDa

50kDa

62kDa

80kDa

40kDa

140kDa

115kDa

240kDa

40kDa

40kDa

**Figure 3B-p-JAK2**

16kDa

30kDa

37kDa

52kDa

66kDa

95kDa

130kDa

175kDa

270kDa

131kDa

131kDa

131kDa

**Figure 3B--JAK2**

131kDa

6.5kDa

15kDa

27kDa

31kDa

50kDa

62kDa

80kDa

115kDa

140kDa

240kDa

**Figure 3B-p-JAK3**

16kDa

30kDa

37kDa

52kDa

66kDa

95kDa

130kDa

175kDa

270kDa

125kDa

125kDa

125kDa

**Figure 3B-JAK3**

125kDa

6.5kDa

15kDa

27kDa

31kDa

50kDa

62kDa

80kDa

115kDa

140kDa

240kDa

**Figure 3B-p-STAT3**

240kDa

140kDa

115kDa

50kDa

88kDa

27kDa

31kDa

62kDa

80kDa

88kDa

88kDa

**Figure 3B-STAT3**

88kDa

6.5kDa

15kDa

27kDa

31kDa

50kDa

62kDa

80kDa

115kDa

140kDa

240kDa

**Figure 3B-GAPDH**

15kDa

27kDa

31kDa

50kDa

62kDa

80kDa

115kDa

140kDa

240kDa

36kDa

36kDa

36kDa

**Figure 3C-Vimentin**

15kDa

27kDa

31kDa

50kDa

62kDa

80kDa

115kDa

140kDa

240kDa

54kDa

54kDa

54kDa

**Figure 3C-E-Cadherin**

16kDa

30kDa

37kDa

52kDa

66kDa

95kDa

130kDa

175kDa

270kDa

130kDa

130kDa

130kDa

**Figure 3C-N-Cadherin**

16kDa

30kDa

37kDa

52kDa

66kDa

95kDa

130kDa

175kDa

270kDa

100kDa

100kDa

100kDa

**Figure 3C-GAPDH**

95kDa

6.5kDa

16kDa

30kDa

37kDa

52kDa

66kDa

36kDa

36kDa

36kDa

**Figure 3D-Bax**

27kDa

21kDa

15kDa

31kDa

50kDa

62kDa

80kDa

115kDa

140kDa

240kDa

21kDa

21kDa

**Figure 3D-BCL2**

16kDa

30kDa

37kDa

52kDa

66kDa

95kDa

130kDa

175kDa

270kDa

26kDa

26kDa

26kDa

**Figure 3D-Caspase-3**

6.5kDa

15kDa

27kDa

31kDa

50kDa

62kDa

80kDa

115kDa

140kDa

240kDa

35kDa

35kDa

35kDa

**Figure 3D-Caspase-9**

6.5kDa

16kDa

30kDa

37kDa

52kDa

66kDa

95kDa

130kDa

175kDa

270kDa

37kDa

46kDa

37kDa

46kDa

37kDa

46kDa

**Figure 3D-TNF-α**

6.5kDa

16kDa

30kDa

37kDa

52kDa

66kDa

95kDa

130kDa

175kDa

270kDa

25kDa

25kDa

25kDa

**Figure 3D-IL-1β**

6.5kDa

16kDa

30kDa

37kDa

52kDa

66kDa

95kDa

130kDa

175kDa

270kDa

17kDa

35kDa

17kDa

35kDa

17kDa

35kDa

**Figure 3D-IL-6**

6.5kDa

16kDa

30kDa

37kDa

52kDa

66kDa

95kDa

130kDa

175kDa

270kDa

23kDa

23kDa

23kDa

**Figure 3D-GAPDH**

6.5kDa

16kDa

30kDa

37kDa

52kDa

66kDa

95kDa

130kDa

175kDa

270kDa

36kDa

36kDa

36kDa

**Figure 4E-CXCL9**

2.7kDa

4.2kDa

6.5kDa

10kDa

15kDa

20kDa

25kDa

30kDa

40kDa

14kDa

14kDa

14kDa

**Figure 4E-CXCR3**

6.5kDa

15kDa

27kDa

31kDa

50kDa

62kDa

80kDa

115kDa

140kDa

240kDa

40kDa

40kDa

40kDa

**Figure 4E-p-STAT3**

16kDa

30kDa

37kDa

52kDa

240kDa

140kDa

115kDa

80kDa

62kDa

88kDa

88kDa

88kDa

**Figure 4E-STAT3**

**Figure 4E-Vimentin**

240kDa

140kDa

115kDa

80kDa

62kDa

50kDa

6.5kDa

15kDa

27kDa

31kDa

54kDa

54kDa

54kDa

**Figure 4E-E-Cadherin**

16kDa

30kDa

37kDa

52kDa

66kDa

95kDa

130kDa

175kDa

270kDa

130kDa

130kDa

130kDa

**Figure 4E-GAPDH**

270kDa

175kDa

130kDa

95kDa

66kDa

52kDa

37kDa

30kDa

16kDa

6.5kDa

36kDa

36kDa

36kDa

**Figure 7A-VEGFA**

270kDa

175kDa

130kDa

95kDa

66kDa

52kDa

37kDa

30kDa

16kDa

23kDa

23kDa

23kDa

**Figure 7A-VEGFR2**

15kDa

27kDa

31kDa

50kDa

62kDa

80kDa

115kDa

140kDa

240kDa

210kDa

210kDa

210kDa

**Figure 7A-GAPDH**

270kDa

175kDa

130kDa

95kDa

66kDa

52kDa

37kDa

30kDa

16kDa

36kDa

36kDa

36kDa

**Figure 7C-p-STAT3**

270kDa

175kDa

130kDa

95kDa

16kDa

30kDa

37kDa

52kDa

66kDa

88kDa

88kDa

88kDa

**Figure 7C-STAT3**

6.5kDa

15kDa

27kDa

31kDa

50kDa

62kDa

80kDa

115kDa

140kDa

240kDa

88kDa

**Figure 7C-p-JAK3**

16kDa

30kDa

37kDa

52kDa

66kDa

95kDa

130kDa

175kDa

270kDa

125kDa

125kDa

125kDa

**Figure 7C-JAK3**

6.5kDa

15kDa

27kDa

31kDa

50kDa

62kDa

80kDa

115kDa

140kDa

240kDa

125kDa

**Figure 7C-p-JAK2**

16kDa

30kDa

37kDa

52kDa

66kDa

95kDa

130kDa

175kDa

270kDa

131kDa

131kDa

131kDa

**Figure 7C-JAK2**

6.5kDa

vv

15kDa

vv

27kDa

vv

31kDa

vv

50kDa

vv

62kDa

vv

80kDa

vv

115kDa

140kDa

240kDa

131kDa

**Figure 7C-GAPDH**

36kDa

36kDa

270kDa

175kDa

130kDa

95kDa

66kDa

52kDa

37kDa

30kDa

16kDa

6.5kDa

36kDa

**Figure 8A-N-Cadherin**

6.5kDa

16kDa

30kDa

37kDa

52kDa

66kDa

95kDa

130kDa

175kDa

270kDa

100kDa

100kDa

100kDa

**Figure 8A-E-Cadherin**

6.5kDa

16kDa

30kDa

37kDa

52kDa

66kDa

95kDa

130kDa

175kDa

270kDa

130kDa

130kDa

130kDa

**Figure 8A-Vimentin**

6.5kDa

15kDa

27kDa

31kDa

50kDa

62kDa

80kDa

115kDa

140kDa

240kDa

54kDa

54kDa

54kDa

**Figure 8A-GAPDH**

6.5kDa

16kDa

30kDa

37kDa

52kDa

66kDa

95kDa

130kDa

175kDa

270kDa

36kDa

36kDa

36kDa

**Figure 8B-Bax**

6.5kDa

15kDa

27kDa

31kDa

50kDa

62kDa

80kDa

115kDa

140kDa

240kDa

21kDa

21kDa

21kDa

**Figure 8B-BCL-2**

27kDa

6.5kDa

15kDa

31kDa

50kDa

62kDa

80kDa

115kDa

140kDa

240kDa

26kDa

26kDa

26kDa

**Figure 8B-Caspase3**

6.5kDa

15kDa

27kDa

31kDa

50kDa

62kDa

80kDa

115kDa

140kDa

240kDa

35kDa

35kDa

35kDa

**Figure 8B-Caspase9**

16kDa

30kDa

37kDa

52kDa

66kDa

95kDa

130kDa

175kDa

270kDa

37kDa

37kDa

46kDa

46kDa

37kDa

46kDa

**Figure 8B-TNF-α**

16kDa

30kDa

37kDa

52kDa

66kDa

95kDa

130kDa

175kDa

270kDa

25kDa

25kDa

25kDa

**Figure 8B-IL-Iβ**

6.5kDa

16kDa

30kDa

37kDa

52kDa

66kDa

95kDa

130kDa

175kDa

270kDa

17kDa

17kDa

35kDa

35kDa

17kDa

35kDa

**Figure 8B-IL-6**

16kDa

30kDa

37kDa

52kDa

66kDa

95kDa

130kDa

175kDa

270kDa

23kDa

23kDa

23kDa

**Figure 8B-GAPDH**

6.5kDa

16kDa

30kDa

37kDa

52kDa

66kDa

95kDa

130kDa

175kDa

270kDa

36kDa

36kDa

36kDa

**Table S1. Statistical data of the top 20 prognostic genes from the 44-gene ccRCC signature.**

This table contains all relevant statistical metrics of the 20 key prognostic genes screened out of the 44-gene ccRCC signature.

**Table S2. Clinicopathological characteristics of 20 ccRCC patients with paired tumor and adjacent normal renal tissues**

This table records complete baseline clinicopathological information of 20 patients with matched tumor and adjacent normal renal specimens.
